# A mixed method approach to analysing patterns and drivers of antibiotic use and resistance in beef farms in Argentina

**DOI:** 10.3389/fvets.2024.1454032

**Published:** 2024-11-13

**Authors:** Cherrill Bedford, Maria Laura Galotta, Georgios Oikonomou, Guadalupe de Yaniz, Matías Nardello, Sergio Sánchez Bruni, Peers Davies

**Affiliations:** ^1^Department of Livestock and One Health, Institute of Infection, Veterinary and Ecological Sciences, University of Liverpool, Liverpool, United Kingdom; ^2^Laboratorio de Farmacología, Facultad de Ciencias Veterinarias, Universidad Nacional del Centro de la Provincia de Buenos Aires-Centro de Investigación Veterinaria Tandil (CIVETAN)-CONICET, Tandil, Argentina

**Keywords:** antibiotic use, antibiotic resistance, feedlots, beef farms, bovine respiratory disease, Argentina, qualitative research

## Abstract

**Introduction:**

Antimicrobial resistance is a challenge to be faced by all livestock sectors; within beef farming, antibiotic use patterns vary by country and management practices. Argentina is a country with high beef production & consumption but limited information surrounding antibiotic use. The aims of this project was to understand how antibiotics are being used across the beef industry in Argentina and exploring drivers of usage.

**Methods:**

Quantitative and qualitative data was collected by: A survey of breeding and feedlot farms including antibiotic use (from purchase data); a detailed analysis of two feedlot farms’ therapeutic antibiotic use records; a survey of vets’ views on certain antibiotic practices; and a focus group of farmers and vets focusing on wider influences affecting decision making. Antibiotic use data was calculated using mg/population corrected unit (PCU) (ESVAC) and thematic analysis was used to identify drivers of antibiotic use among participants.

**Results:**

The median use across 17 farms that supplied purchase data was 76.52  mg/kg PCU (ESVAC; IQR  =  36.81  mg/kg PCU [ESVAC]). The detailed farm records showed that the largest reason for treatment was group treatments (72.92% of treatments) followed by treatment for respiratory disease (12.75% of treatments). Macrolides accounted for 76.37% of treatments. Nearly half of farms used routine prophylactic treatment for arriving animals (n  =  7/18). The use of quarantine and ‘sick pens’ were seen as important by surveyed vets with antibiotic prophylaxis and in-feed antibiotics seen as contributors to antibiotic resistance. The focus group highlighted the influence of the economic and political landscape on husbandry practices and the responsibility the farming sector had towards antibiotic stewardship.

**Discussion:**

Overall, Argentine beef feedlots resemble North American beef feedlots in terms of antibiotic practices but with considerably lower usage, with in-feed monensin representing a large proportion of total ABU. The adaptation period presents a challenge to animal health; antibiotics are administered a prophylaxis, metaphylactic and individual treatments depending on farm management practices. Further research into internationally comparable measures of ABU and detailed cost-benefit analysis of practical, on-farm interventions are needed to aid improved antimicrobial stewardship in livestock systems globally.

## 1 Introduction

Antimicrobial resistance (AMR), especially antibiotic resistance (ABR), is a worldwide problem across many sectors including human and animal healthcare and as such requires a multidisciplinary approach to improve outcomes (1). ABR threatens public health not only via increased human mortality and morbidity through multi-drug resistance diseases (4.95 million deaths worldwide in 2019) (2) but also by threatening food security (3). While the mechanisms linking increased antibiotic use (ABU) in animals to increased human morbidity and mortality are not yet fully documented (4) it is widely accepted that improving antibiotic stewardship and decreasing use within the food-producing animal sector is an important part of the solution for tackling ABR (5, 6).

Mulchandani et al. (7) estimated that in 2020 the food-producing animal sector was responsible for the use of 99,502 tonnes of active antimicrobial ingredient and this is estimated to rise to 107,472 tonnes in 2030. Ardakani et al. (8) estimated using data from 2019 to 2021 that cattle were responsible for 53.3% of this global total. Antibiotics are used in the beef sector for a range of reasons (9). International comparisons of use within the beef sector are difficult due to the sparsity of published data and the range of incompatible methods used for estimating use (10, 11). Within the grey literature The Alliance to Save Our Antibiotics (12) reports 15 to 25 mg/kg used in cattle in the United Kingdom compared to 237 mg/kg in cattle in the United States.

In 2015 the Ministry of Health and the Ministry of Agriculture, Livestock and Fisheries in Argentina published a strategy for the control of AMR including recommendations for increased vigilance towards AMR in both humans and animals (13). However, Prack McCormick et al. (14) reported that their study “gives evidence for supporting the hypothesis that AMR of common food-transmitted bacteria in Argentina is reaching alarming levels.” In 2019 there were 9,000 deaths in Argentina directly attributable to AMR and 35,300 associated with AMR (15). A scoping review on AMR in rural Latin America used two papers from Argentina (9.5%) and identified research gaps on the drivers of AMR in Latin America with regard to data on AMU in livestock farming and the link to environmental reservoirs of resistance (16).

Argentina has the highest *per capita* beef consumption in the world at 52.2 kg/person/year (17). The Argentine beef herd in 2020 comprised 52.91 million head of cattle across more than 200,000 farming businesses (18). However, research into ABU and ABR within the beef sector is lacking. Prack McCormick et al.’s (14) study identified a dearth of research within the field of beef feed-lots. A study of beef and dairy farms in Santa Fe province, Argentina, reported that 74.6% of survey respondents believed that AMR was making it harder to treat sick animals and 51.4% believed that AMR in humans is linked to AMU in food-producing animals (19). Further research into AMU and AMR in Argentina is currently at a preliminary stage, early findings from Safar et al. (20) of a survey for Argentinian veterinarians suggest “98.9% of those surveyed consider that AMR is an important or very important problem and of interest to the world” though less than 20% of those surveyed were large animal vets.

The situation regarding ABU within the beef sector in Argentina needs to be viewed through the lens of the economic and political situation within the country as this can have major implications for farm businesses. In May 2021, the then government banned the export of nearly all beef products from Argentina in an attempt to keep domestic prices low and to slow down inflation (21). In the year prior to this ban Argentina exported 929,000 tonnes of beef worth around USD 2.7 billion with over 75% of these exports to China (22). In addition to export restrictions, prices for the most popular beef cuts were reduced by around 30% by the Economy Ministry in February 2023 (23). Despite these measures inflation continued to rise hitting 160.9% in November 2023; the data collection phase of this study (24).

Qualitative data is an important, and often underused, tool in understanding the attitudes and perceptions driving ABU and how behaviour change interventions could be successfully implemented (25). In an under-researched area, such as ABU in Argentina, qualitative data adds depth and breadth of understanding and allows for previously undisclosed issues to be raised and explored.

The primary aim of this study was to investigate the current attitudes and perceptions towards ABU and ABR within the beef sector in Argentina with the objective of answering the following questions:

- How are antibiotics currently being used across the beef sector in Argentina?
- What are the factors that currently drive usage?
- What is the importance of farmer and veterinary knowledge and attitudes as drivers of ABU?

## 2 Materials and methods

**Figure 1:**
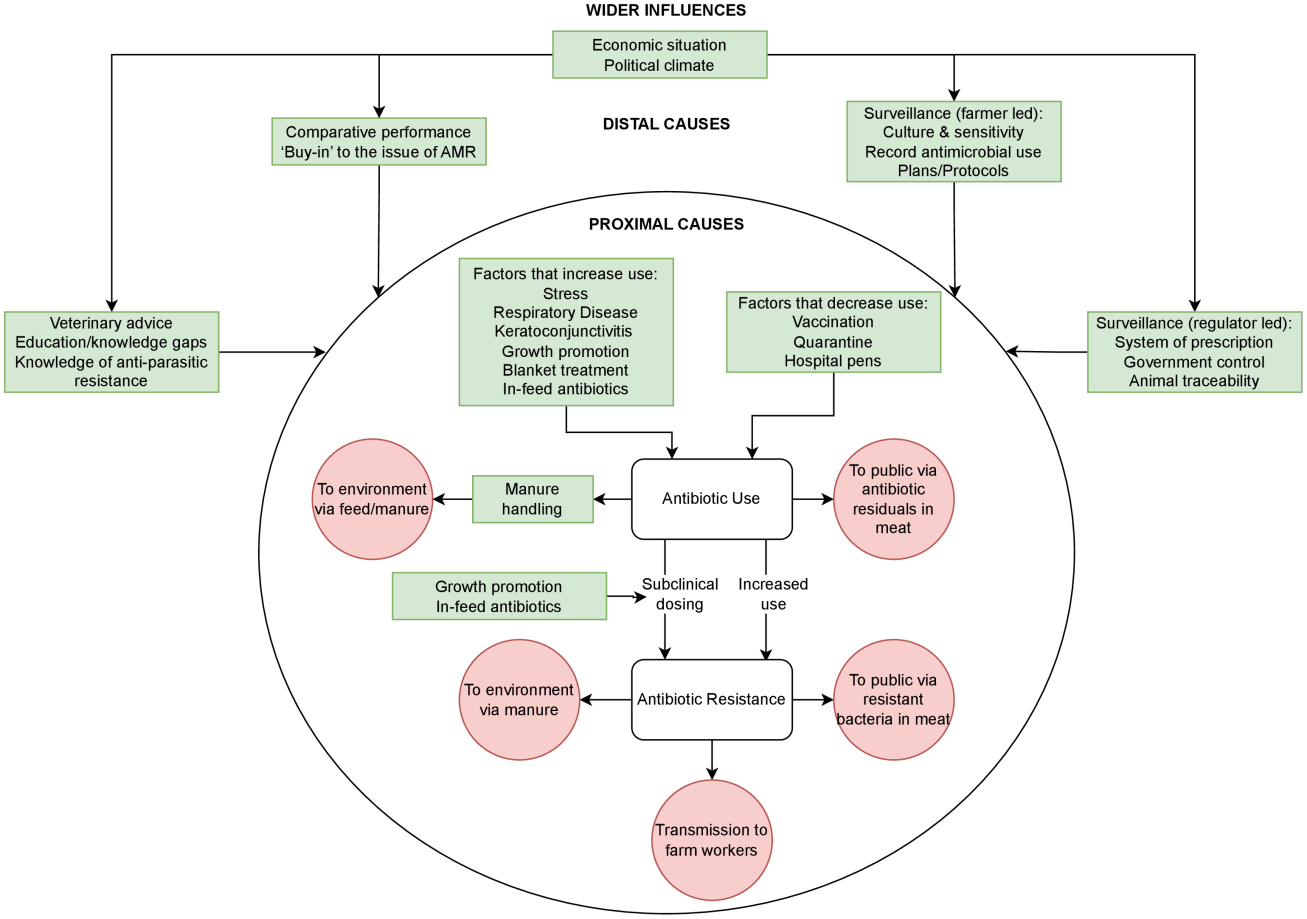
Conceptual framework of coded themes (green) showing their relationship to each other (arrows) and known resistance pathways (red).

A conceptual framework was developed to map out the independent variables (drivers) of antibiotic use and antibiotic resistance (dependent variables) in the Argentine beef sector by a combination of literature review and expert opinion from the members of the research team. The purpose of the framework being to assist in the identification of specific areas of data collection within the subjectivist inductive research approach, as described by Varpio et al., into the distal and proximal drivers of the dependent variables (4, 26–28) as they operate in the Argentine context (Figure 1).

A sequential explanatory design sequential explanatory design mixed methods approach was adopted to explore the patterns of antibiotic usage, in terms of quantities and classes of products, along with an exploration of the perceptions of antibiotic use held by farmers and veterinarians, with quantitative data collection on antibiotic usage by antibiotic class and temporal patterns of use over the production cycle supporting the involvement of specific independent variables, such a pneumonia treatment, which were then focused upon in the subsequent qualitative data collection phases. This combination of quantitative and qualitative methodologies allows antibiotic use in the Argentine beef sector to be compared with existing data from and other cattle sectors globally. This analysis then informed the interview and focus group topics addressed in the qualitative data collection. This provided an equally novel insight into the motivations and drivers of antibiotic use in this livestock industry and geographic region which have hitherto been largely neglected in the published literature. Four different data collection methodologies were used during the period March 2020 to November 2023: a survey of farms including antibiotic use from purchase data (ABU SURVEY); a convenience sample of two farms’ therapeutic antibiotic use (TREATMENT RECORDS); a survey of vets’ views on certain antibiotic practices (KAA SURVEY); and a focus group of farmers and vets focusing on wider influences affecting decision making (FOCUS GROUP). These are explained in detail below (ABU SURVEY).

Farms were recruited via advertisement on the website of the Argentine Feedlot Chamber (CAF) and through veterinarians affiliated to the network of the National Institute of Agricultural Technology (INTA). Participating farms were situated in four provinces: Buenos Aires Province, Chubut, Chaco, and Córdoba.

18 farms were recruited and visited in person by the same researcher, ABU data as well as farm management data was collected by way of a researcher-assisted survey (Supplementary Document 1 and 2), the survey included open ended questions with free text responses. Antibiotic purchase data was collected by self-reported purchase history for a minimum of the preceding 12 months, annualised and calculated using average animal numbers per farms to give mg/population corrected unit (PCU) using the European Surveillance of Veterinary Antimicrobial Consumption method (ESVAC). Usage of in-feed antibiotics including monensin was calculated from the ration in mg/kg PCU (ESVAC); farms that did not supply data on monensin were excluded from monensin specific calculations. The ESVAC PCU figure for beef cattle of 425 kg was used throughout (29).

All statistical analyses were performed using R Statistical Software (v4.3.1) (30) with the *Tidyverse* (31) and *Stats* (30). The “*Stats*” package was used to perform independent two-sample Welch’s t-tests comparing antibiotic use across differing management practices. A significance value of *p* < 0.05 was used to determine statistically significant results (Supplementary Table 1).

### 2.1 Treatment records

In addition to antibiotic purchase data, a convenience sample of two farms provided detailed individual animal and group antibiotic treatment records, including reason for treating, number of animals treated, and medication used. This data was downloaded from their electronic medicine recording system and used for descriptive statistical analysis.

### 2.2 KAA survey

An online survey (Supplementary Document 3 and 4; in Castellano Spanish) was developed and advertised to veterinarians who worked within the beef sector in Argentina via the Veterinary College of Buenos Aires Province (CVPBA) and the Argentinian Federation of Veterinarians (FEVA). 19 veterinarians completed the survey.

Following analysis of the ABU data collected earlier in the project, BRD was identified as a major causative factor of ABU; as a consequence, BRD was used as the focus for the survey with questions relating certain inventions to effectiveness in combating BRD and their impact on ABU/ABR. The survey comprised:

- Likert style questions of agreement to various statements (the responses were from left to right: Totally agree, Agree, Neutral, Disagree, Totally disagree) (32). Responses in each of the agreement categories were calculated as percentages and displayed in Likert charts.
- Rank-order questions focusing on the prevention and treatment of BRD with a view to antibiotic stewardship. Ranks were summed and shown in histograms, median and interquartile range (IQR) values were also reported.
- Two questions asked for numeric data, data was summarised in box plots showing the median, IQR and range.
- The survey also allowed for free text responses.
- After meeting the enrolment criteria (a qualified veterinarian with responsibility for at least one beef farm) and consenting to take part in the survey all further questions were optional to improve response rate (by minimising the drop-off rate).
- The following terms were defined: prophylaxis as the treatment of a group of animals to prevent disease without the presence of symptoms; metaphylaxis as the treatment of a group of animals with a proportion of the group showing symptoms.

### 2.3 Focus group

Preliminary interviews and discussions with farmers and veterinarians in both Buenos Aires province and Tandil were used to create the discussion guide for the focus group.

An in-person focus group was held in Chubut province composed of farmers and veterinarians associated with, and recruited via, a single veterinary practice in Chubut that used a convenience sampling strategy and recruited participants via telephone. Eight farmers and two veterinarians attended the focus group which lasted approximately 1 h and was facilitated by a Spanish speaking British researcher with a background in farm animal epidemiology. The participants farmed in close geographic proximity and knew each other prior to the focus group, the focus group was situated at one of the participant’s farm. The participants were male aged between 25 and 65 years old.

Pre-determined questions were introduced to the discussion in such a way as to aid the flow of conversation and build on topics raised independently by the group using a semi-structured discussion guide (Supplementary Document 5 and 6). The focus group was audio recorded, and notes were taken during the discussion. The transcript was transcribed, cleaned, and translated into English by the researcher with the translation of unknown terms checked with Argentinian researchers associated with the project.

### 2.4 Qualitative data analysis

Free text from ABU SURVEY, KAA SURVEY and the transcript from FOCUS GROUP were coded by a single data coder using QDA Miner Lite software (v3.0) (33). An inductive thematic approach to coding was used building on common themes that recurred across the methodologies (34), sub themes were grouped into four broader themes based on proximity to the decision-making process around antibiotic use. Quotes have been edited for clarity: text in square brackets, e.g., [dose], have been added; ellipses (…) indicate text removed. Identifiable names, businesses and some place names have been redacted, e.g., [name], to maintain confidentiality.

## 3 Results

A conceptual framework encompassing ABU, ABR and resistance pathways in beef farms was developed using previous literature, expert opinion and themes from qualitative data analysis (4, 26–28) (Figure 1).

**Figure 2:**
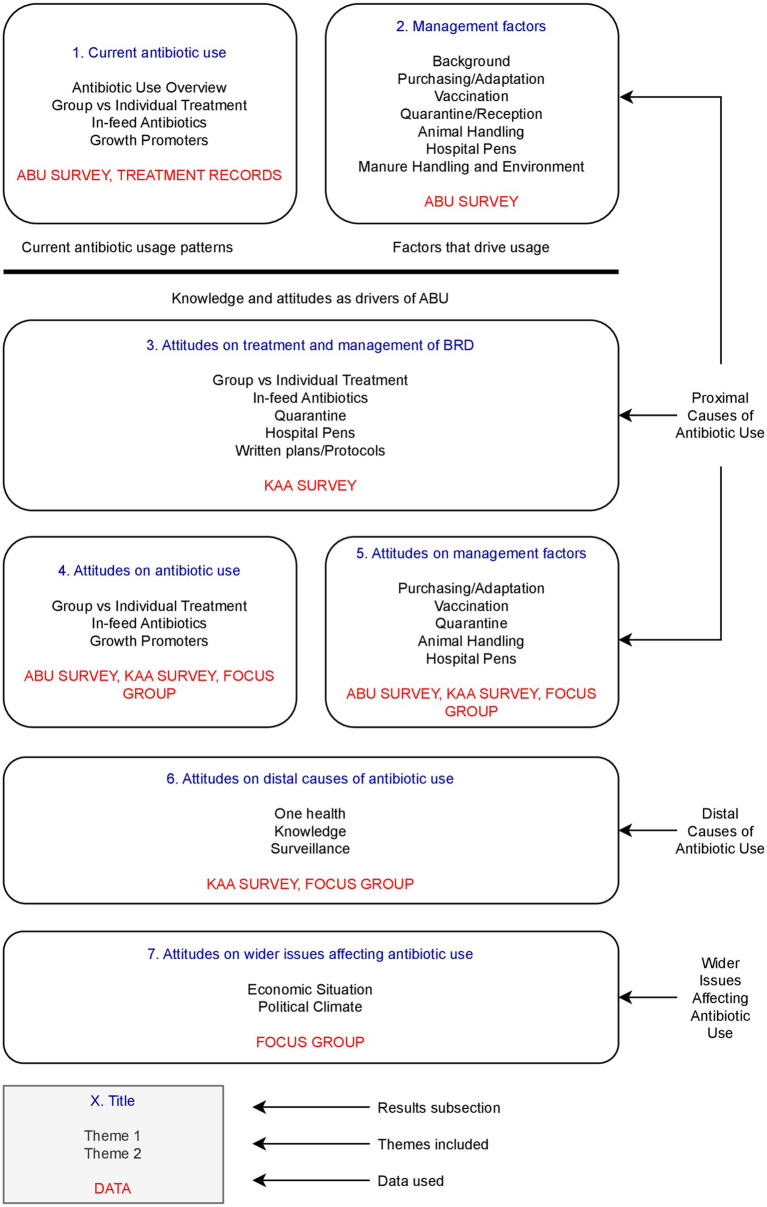
Infographic showing the subsections within the results, the themes discussed, and data sources used. ABU SURVEY = Survey regarding antibiotic use and management practices of 18 farms. TREATMENT RECORDS = Survey regarding detailed antibiotic use and treatment records of 2 farms. KAA SURVEY = Survey regarding attitudes on treatment and management for BRD of 19 vets. FOCUS GROUP = Focus group of 8 farmers and 2 vets.

Data was analysed by topic and data collection method (Figure 2).

### 3.1 Current antibiotic use

Data used: ABU SURVEY, TREATMENT RECORDS.

**Figure 3:**
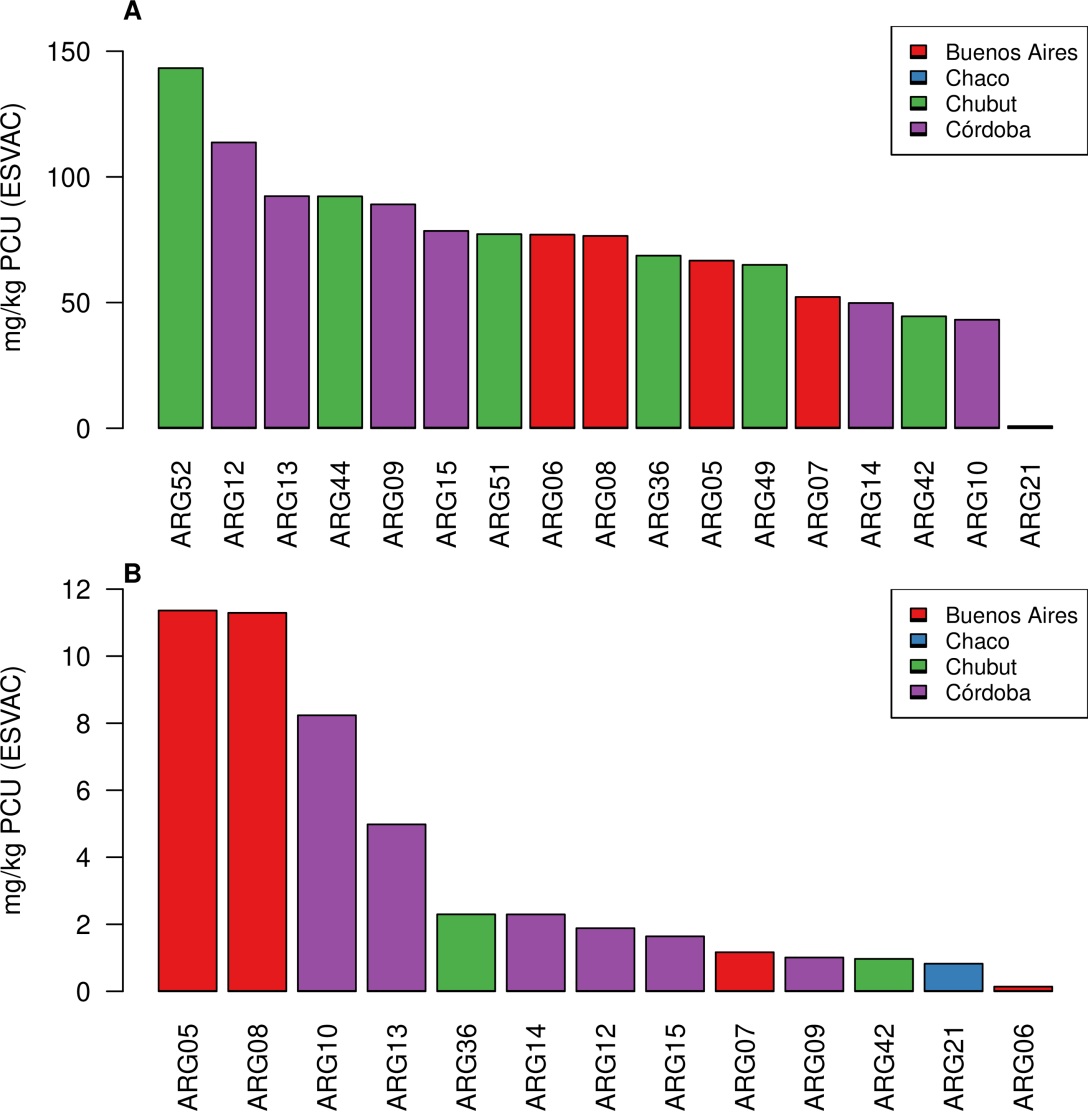
Bar plots showing antibiotic use in mg per population corrected units (PCU) using the European Surveillance of Veterinary Antimicrobial Consumption method (ESVAC). **(A)** Shows total antibiotic use. **(B)** Shows antibiotic use excluding monensin. Colours represent farms’ provinces in Argentina.

Antibiotic data collected via self-reported purchase data and calculated into mg/kg PCU (ESVAC) was analysed per farm and per region. Seventeen farms from four regions were analysed; the highest farm reported ABU totalling 143.26 mg/kg PCU (ESVAC); the lowest farm reported 0.82 mg/kg PCU (ESVAC). The median use across these 17 farms was 76.52 mg/kg PCU (ESVAC; IQR = 36.81 mg/kg PCU [ESVAC]). Monensin was removed from the dataset and the same calculations were undertaken. 13 of the 17 farms had records that included antibiotics other than monensin; ABU figures ranged from 11.36 to 0.14 mg/kg PCU (ESVAC) with a median of 1.88 mg/kg PCU (ESVAC; IQR = 3.97 mg/kg PCU [ESVAC]; Figure 3).

**Figure 4:**
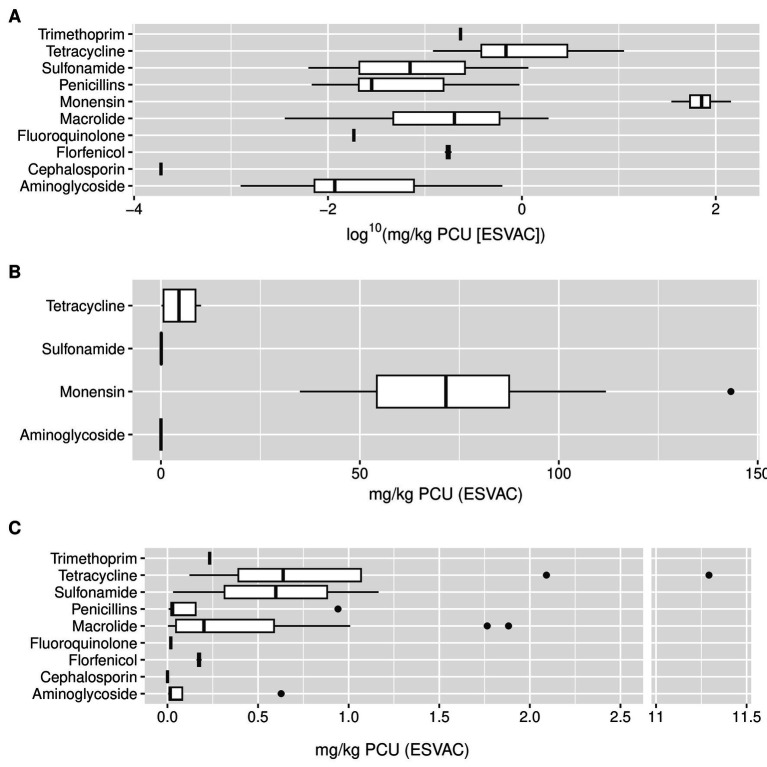
Barplots showing antibiotic use by antibiotic type in mg per population corrected units (PCU) using the European Surveillance of Veterinary Antimicrobial Consumption method (ESVAC). **(A)** log^10^ scale plot of total antibiotic use (*n* = 17 farms). **(B)** Oral route antibiotics (*n* = 17 farms). **(C)** Injection route antibiotics (*n* = 11 farms). Box shows lower quartile (Q1), median (in bold) and upper quartile (Q3), whiskers show 1.5 the interquartile range (IQR) below or above Q1/Q3, respectively, (i.e., Q1–1.5 * IQR and Q3 + 1.5 * IQR). Outliers outside the whiskers are shown as data points. One outlier for tetracycline at 11.29 mg/kg PCU (ESVAC) was removed from this analysis to avoid the figure becoming excessively compressed.

This ABU data from 17 farms was analysed by antibiotic type. Ten different antibiotic types were identified with monensin showing the highest median use 71.63 mg/kg PCU (ESVAC; IQR = 36.28 mg/kg PCU [ESVAC], *n* = 17) and cephalosporin showing the lowest median use 0.00019 mg/kg PCU (ESVAC; *n* = 1). Four oral route antibiotics were identified with monensin showing the highest use as before, other oral antibiotic types included tetracycline, sulfonamide, and aminoglycoside. Nine injectable antibiotic types were identified with tetracycline showing the highest median use 0.64 mg/kg PCU (ESVAC; IQR = 0.68 mg/kg PCU [ESVAC], *n* = 8) followed by sulfonamide 0.60 mg/kg PCU (ESVAC; IQR = 0.57 mg/kg PCU [ESVAC], *n* = 2; Figure 4).

**Figure 5:**
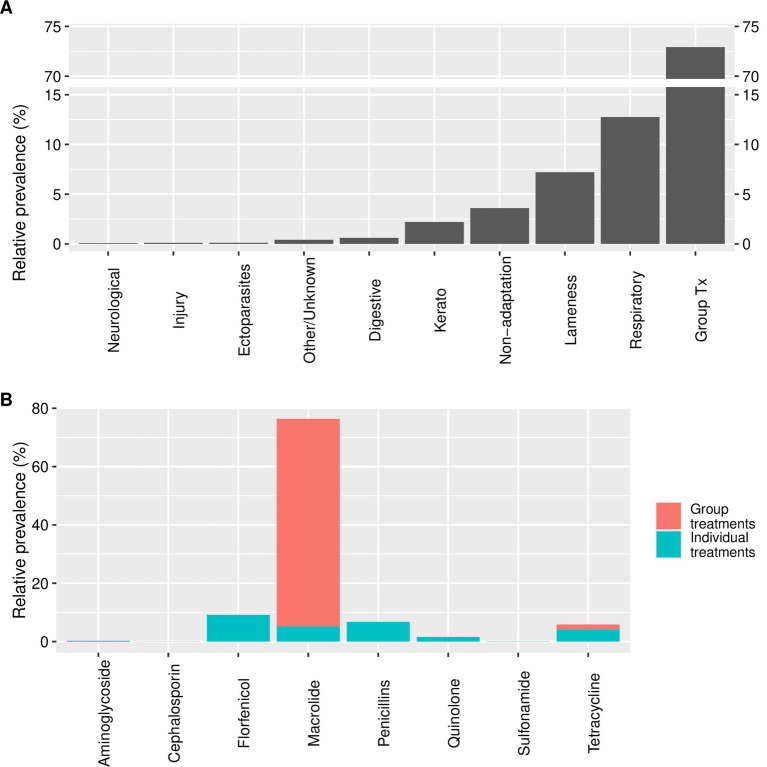
Bar plots showing detailed data from two farms on **(A)** reason for treatment (“Group Tx” includes prophylaxis, metaphylaxis or group disease treatment) and **(B)** antibiotic type used; measured in percent of treatments given.

Two farms supplied detailed treatment records, and the data was analysed according to reason for treatment and antibiotic type. The main reason for treatment was group treatments of an unspecified nature (i.e., including prophylaxis, metaphylaxis or group disease treatment; 72.92% of treatments) followed by treatment for respiratory disease (12.75% of treatments). Macrolides were used the most often accounting for 76.37% of treatment (93.23% of this use was for group treatments). Excluding group treatments Florfenicol was the most used antibiotic type accounting for 33.87% of (non-group) treatments (Figure 5).

Seven farms stated they used routine prophylactic treatment for arriving animals (*n* = 7/18). ABU for farms that used routine prophylactic treatment for arriving animals tended to be lower than those which did not (15%) at 65.43 mg/kg PCU (ESVAC; Standard Deviation (SD) = 27.14 mg/kg PCU [ESVAC]) vs. 77.28 mg/kg PCU (ESVAC; SD = 37.54 mg/kg PCU [ESVAC]) however the sample size was not sufficient to demonstrate if this was statistically significant (*p* = 0.49). With a statistical power of 80%, 82 farms would be needed in each group to determine a statistically significant difference assuming similar means/SD.

**Figure 6:**
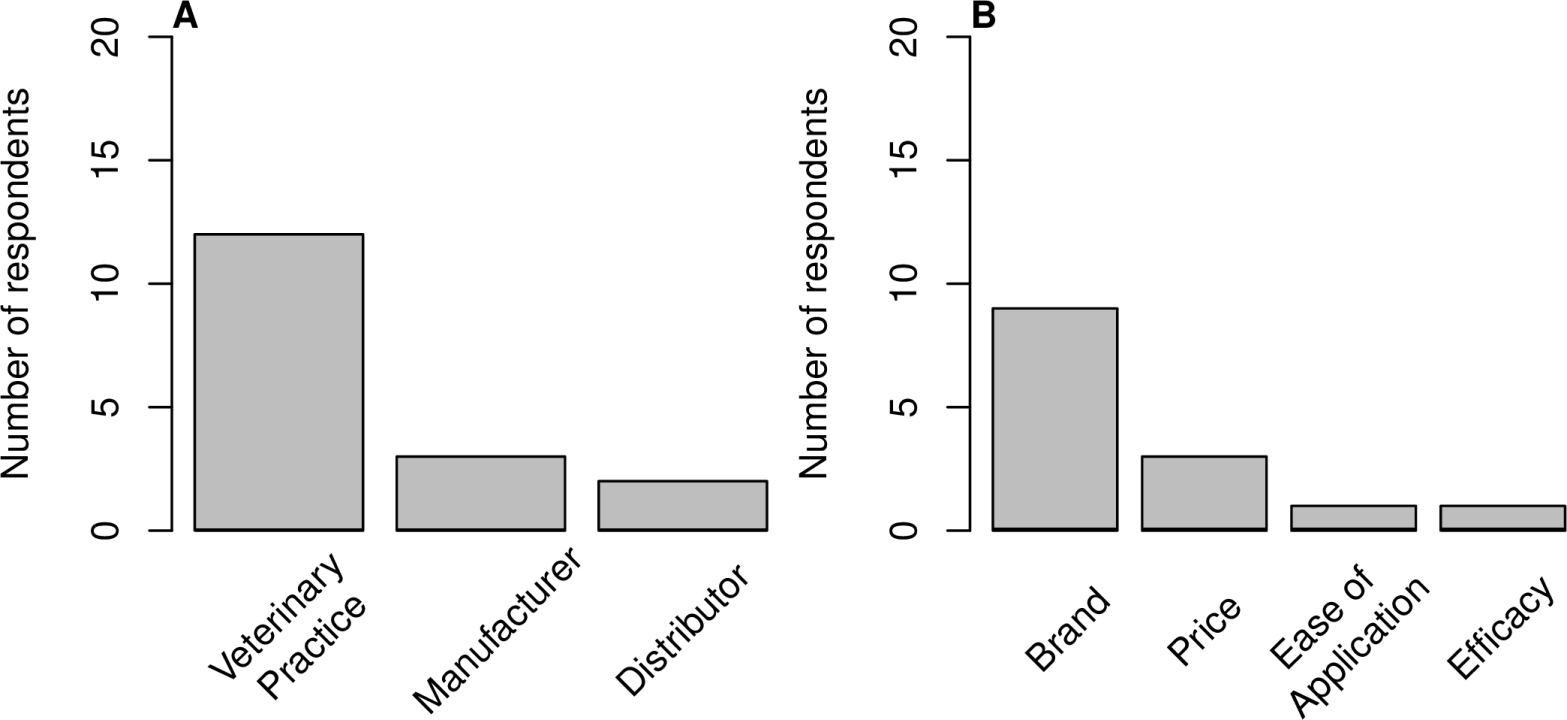
Bar plot showing answers to questions about management procedures, respondents could choose more than one answer. **(A)** Antibiotic vendor used (*n* = 15). **(B)** Influences of antibiotic purchase (*n* = 12).

The majority of farms used antibiotic metaphylaxis either occasionally or regularly (*n* = 10/18). For farms that used antibiotic metaphylaxis (either occasionally or regularly) compared to those that did not ABU figures were 62.05 mg/kg PCU (ESVAC; SD = 28.97 mg/kg PCU [ESVAC]) vs. 84.04 mg/kg PCU (ESVAC; SD = 31.11 mg/kg PCU [ESVAC]); this difference was not statistically significant (*p* = 0.15). The majority of farms relied on veterinarians alone to purchase the antibiotics (*n* = 10/15); they sourced antibiotics from a veterinary practice (*n* = 12/15; Figure 6A) and selected brand as an important factor when choosing antibiotics (*n* = 9/12; Figure 6B).

### 3.2 Management factors

Data used: ABU SURVEY.

#### 3.2.1 Background

Sampled farms came from four provinces of Argentina: Córdoba (*n* = 7/18), Chubut (*n* = 6/18), Buenos Aires Province (*n* = 4/18) and Chaco (*n* = 1/18). The majority of farms solely supplied the domestic market (*n* = 13/18) with the remainder supplying both domestic and international markets (*n* = 5/18).

Farms had solely feedlots (*n* = 11/18); feedlots and hotel fattening systems (*n* = 3/18); feedlots or pasture with supplementation (*n* = 2/18). Two farms only fattened animals on pasture with supplementation (*n* = 2/18).

**Figure 7:**
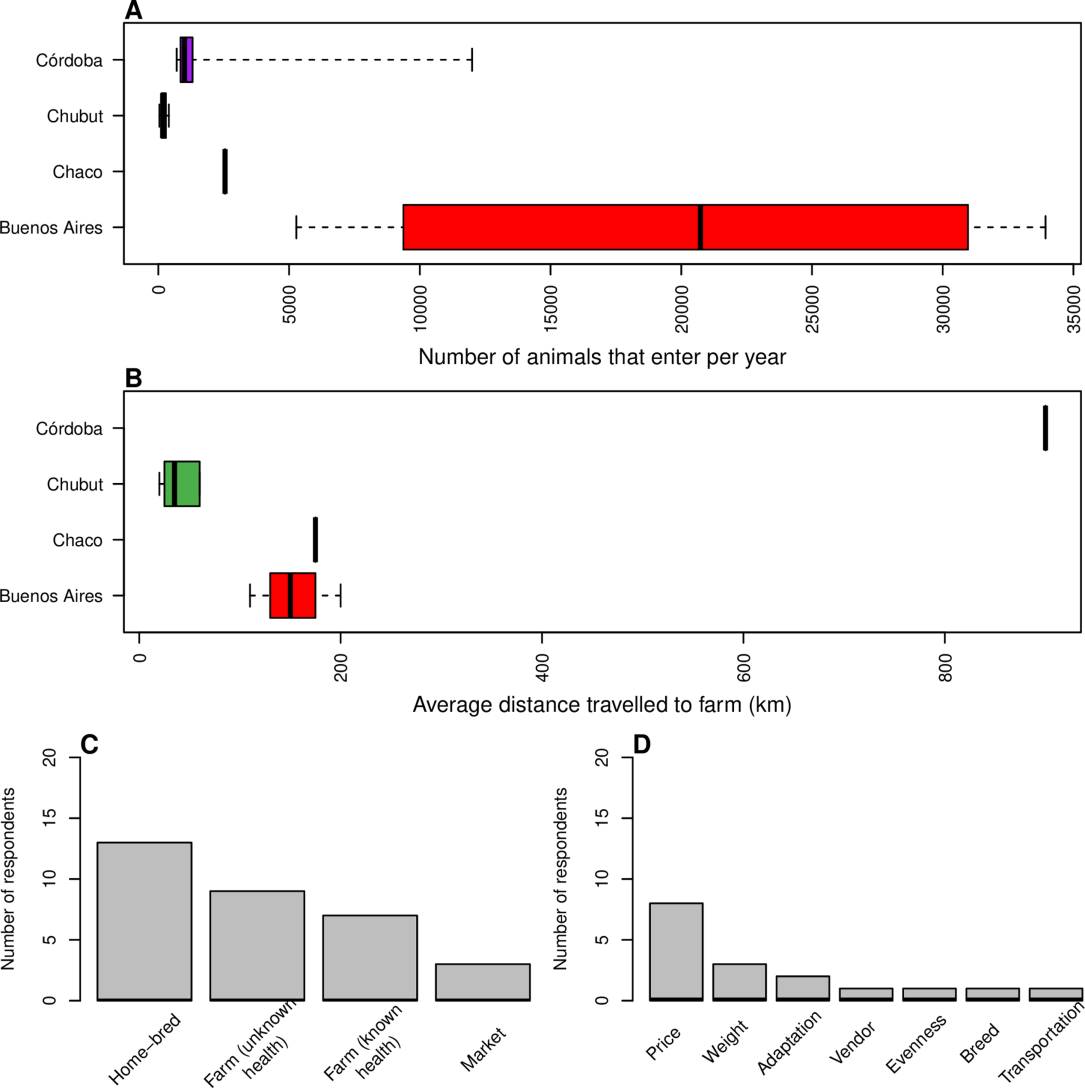
**(A)** Box plots split by province showing numbers of animals that enter the farm each year (*n* = 18). **(B)** Box plots split by province showing average distance (km) that animals travel to enter the farm (*n* = 11). **(A,B)** Box shows lower quartile, median (in bold) and upper quartile, whiskers show the range. **(C,D)** Bar plots showing answers to questions about management procedures, respondents could choose more than one answer. **(C)** Origin of animals for fattening (*n* = 18). **(D)** Reasons for choosing animals to purchase (*n* = 11).

Farms had between 40 animals to over 33,000 entering the farm within the previous 12 months with a mean of 5,690 (SD = 2,370). Farms based in Buenos Aires provinces had the highest median number of animals entering the farm (20,731, IQR = 18,066, *n* = 4/18) followed by Chaco (2,550, *n* = 1/18), Córdoba (1,000, IQR = 460, *n* = 7/18) and Chubut (165, IQR = 135, *n* = 6/18; Figure 7A).

#### 3.2.2 Purchasing/adaptation

A majority of farms sourced at least some of their stock from home-bred animals (*n* = 13/18) and half of the farms sourced animals from farms of unknown health (*n* = 9/18; Figure 7C). Two thirds of farms bought calves (less than 12 months of age, *n* = 12/18) and price was considered to be the most important factor when choosing buying in new stock (*n* = 8/11; Figure 7D).

The average distance animals would travel to enter the farm ranged from 20 km to 900 km with a mean of 161 km (SD = 76 km). Córdoba had the highest median distance (900 km, *n* = 1/11) followed by Chaco (175 km, *n* = 1/11), Buenos Aires Province (150 km, IQR = 45 km, *n* = 3/11) and Chubut (35 km, IQR = 29 km, *n* = 6/11; Figure 7B).

A diversity of purchasing practices was observed, with farmers from three provinces: Buenos Aires Province, Chaco, and Chubut, sourcing animals from within from their own province. While farmers from 1 province, Córdoba, sourced animals from multiple different provincial regions (Buenos Aires Province, Corrientes, Entre Rios, Formosa and San Luis).

Farmers were asked in which period the greatest morbidity/mortality occurs (options: adaptation period, fattening period, finishing period); of the 13 farmers that responded all stated the adaptation period.

#### 3.2.3 Vaccination

Only one farm measured temperature as part of the entry protocol (*n* = 1/17), the majority of farms vaccinated animals upon arrival (clostridial vaccine: *n* = 17/18, respiratory vaccine: *n* = 13/18, keratoconjunctivitis vaccine: *n* = 9/15). All farms applied antiparasitic treatment upon arrival (*n* = 18/18).

#### 3.2.4 Quarantine/reception

**Figure 8:**
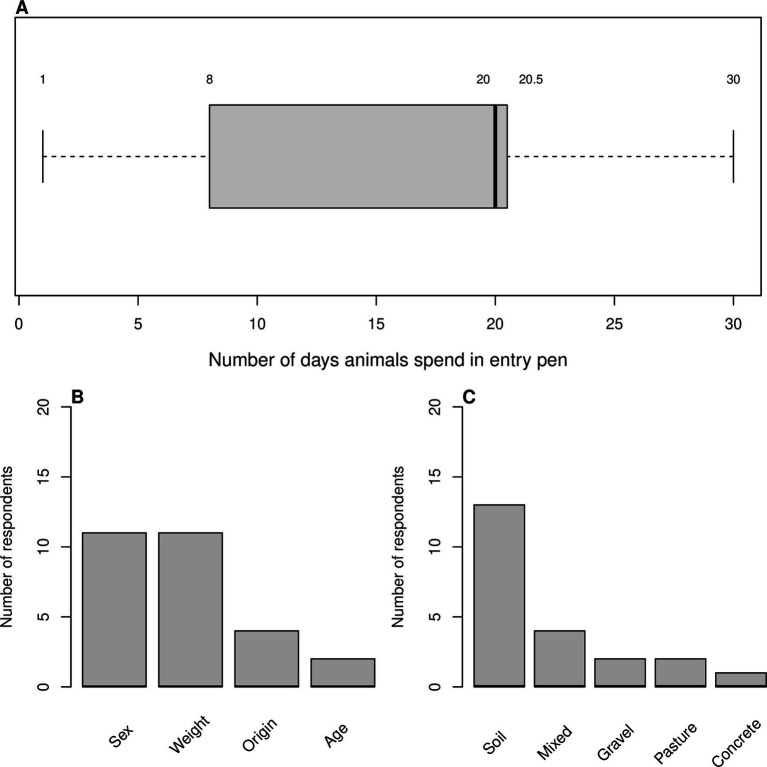
**(A)** Box plot showing number of days animals spend in an entry pen (*n* = 7); box shows lower quartile, median (in bold) and upper quartile, whiskers show the range. **(B,C)** Bar plots showing answers to questions about management procedures, respondents could choose more than one answer. **(B)** Criteria for pen allocation (*n* = 14). **(C)** Substrate of feedlot pen/s (*n* = 18).

Farmers were asked about a range of measures applied as part of the entry protocol; just under half of farms used a reception pen for new animals (*n* = 8/17). ABU for farms that did use a reception pen was numerically lower than those that did not: 70.13 mg/kg PCU (ESVAC; SD = 28.51 mg/kg PCU [ESVAC]) vs. 77.41 mg/kg PCU (ESVAC; SD = 36.62 mg/kg PCU [ESVAC]); this was not statistically significant (*p* = 0.67). Of those that did use an entry pen, the time animals remained in this pen ranged from 1 to 30 days with a median of 20 days (IQR = 12.5 days, *n* = 7; Figure 8A).

#### 3.2.5 Animal handling

In general animals were sorted into pens according to sex and/or weight (*n* = 11/14 for either; Figure 8B) and animals were moved into a more appropriate pen if needed (*n* = 8/15).

#### 3.2.6 Hospital pens

The majority of farms did not use a hospital pen for sick animals (*n* = 11/17).

#### 3.2.7 Manure handling and environment

Only one of the farms had treatment facilities for manure which involved settling pools for liquid waste, solid waste was removed every 4 years and left to compost for a year before being spread on the fields. Other farms reported sloped pens to allow for natural draining but no lagoons to capture the liquid waste, using earth mounds in the pens in wet weather to create a dry space and raking pens between groups. Three farms reported having new feedlots in the last 2–3 years that had not yet been cleaned.

The majority of farms had soil pens (*n* = 13/18; Figure 8C) and sourced their water from a well or borehole (*n* = 9/13).

### 3.3 Attitudes on treatment and management of BRD

Data used: KAA SURVEY.

Survey respondents were asked Likert style questions asking to what extent they agreed with various statements regarding treatment and management approaches to BRD. The overwhelming majority agreed to some extent with the statements:

- “The use of sick pens helps prevent the spread of BRD” (100%, *n* = 19 of 19),
- “Quarantine pens need to be situated away from other pens (i.e., no nose-to-nose contact) to be effective” (100%, *n* = 19 of 19),
- “Quarantining new animals helps prevent the spread of BRD” (95%, *n* = 17 of 18),
- “Written plans/protocols help farms to improve their management of BRD” (95%, *n* = 18 of 19), and
- “Sick pens need to be situated away from other pens (i.e., no nose-to-nose contact) to be effective” (89%, *n* = 17 of 19).

Eighty-eight percent (*n* = 16 of 18) of respondents were either in agreement or neutral about the statement “Animals in the sick pen recover faster than animals treated in group pens.”

**Figure 9:**
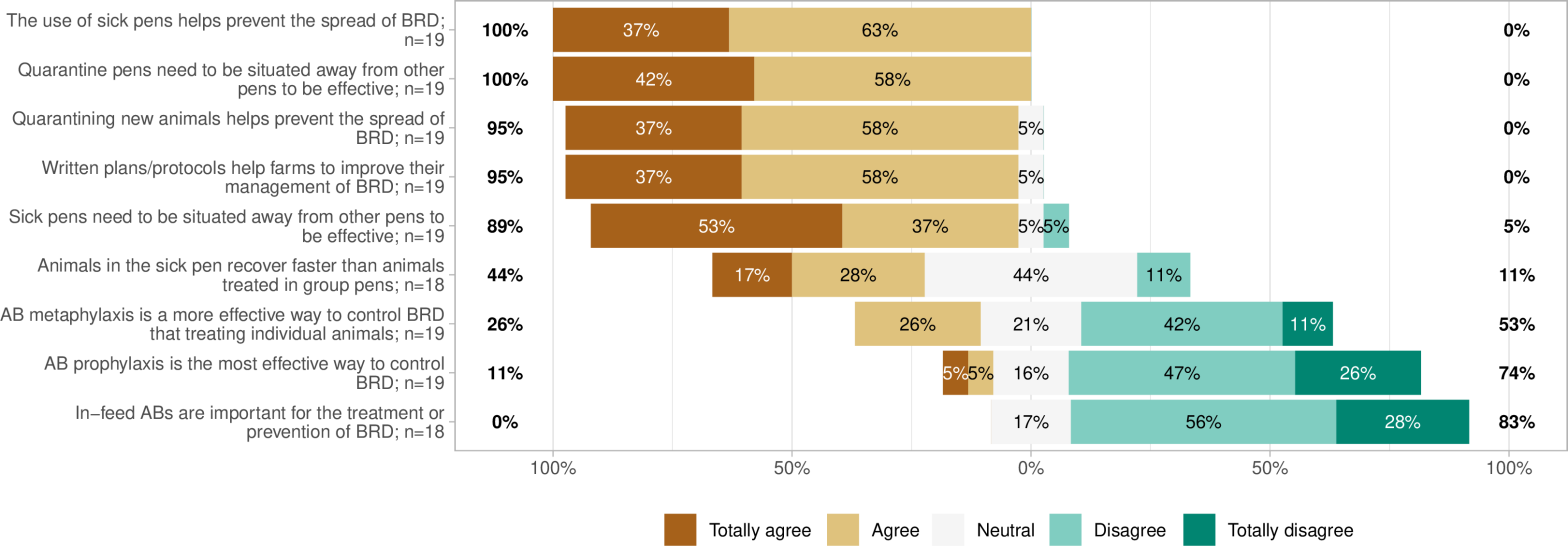
Likert chart showing the response to nine statements regarding treatment and management approaches to bovine respiratory disease (BRD).

The majority disagreed to some extent with the statements:

- “Antibiotic metaphylaxis is a more effective way to control BRD that treating individual animals” (53%, *n* = 10 of 18),
- Antibiotic prophylaxis is the most effective way to control BRD” (74%, *n* = 14 of 19), and
- “In-feed antibiotics are important for the treatment or prevention of BRD” (83%, *n* = 16 of 19; Figure 9).

Survey respondents were also asked Likert style questions asking to what extent they agreed with various statements regarding the effect of treatment and management approaches to BRD on ABR. The majority of respondents agreed to some extent with 12 of the 13 statements:

- “Antibiotic prophylaxis should be avoided where possible” (100%, *n* = 19 of 19).
- “Measuring antibiotic use is an important step to reducing use” (100%, *n* = 18 of 18).
- “Encouraging farmers to move away from antibiotic prophylaxis will help the issue of antibiotic resistance” (95%, *n* = 18 of 19).
- “Encouraging farmers to minimise their use of in-feed antibiotics will help the issue of antibiotic resistance” (94%, *n* = 17 of 18).
- “Using in-feed antibiotics can lead to under-dosing or intermittent dosing” (89%, *n* = 16 of 18).
- “Encouraging farmers to create written plans/protocols will help the issue of antibiotic resistance” (89%, *n* = 17 of 19).
- “Antibiotic metaphylaxis should be avoided where possible (in favour of treating individual animals)” (84%, *n* = 16 of 19).
- “In-feed antibiotics should be avoided where possible” (83%, *n* = 15 of 18).
- “Encouraging farmers to move away from antibiotic metaphylaxis will help the issue of antibiotic resistance” (83%, *n* = 15 of 18).
- “Encouraging farmers to quarantine will help the issue of antibiotic resistance” (74%, *n* = 14 of 19).
- “Encouraging farmers to use sick pens will help the issue of antibiotic resistance” (68%, *n* = 13 of 19).
- “Antibiotic metaphylaxis is easier for farmers than treating individual animals” (61%, *n* = 11 of 18).

**Figure 10:**
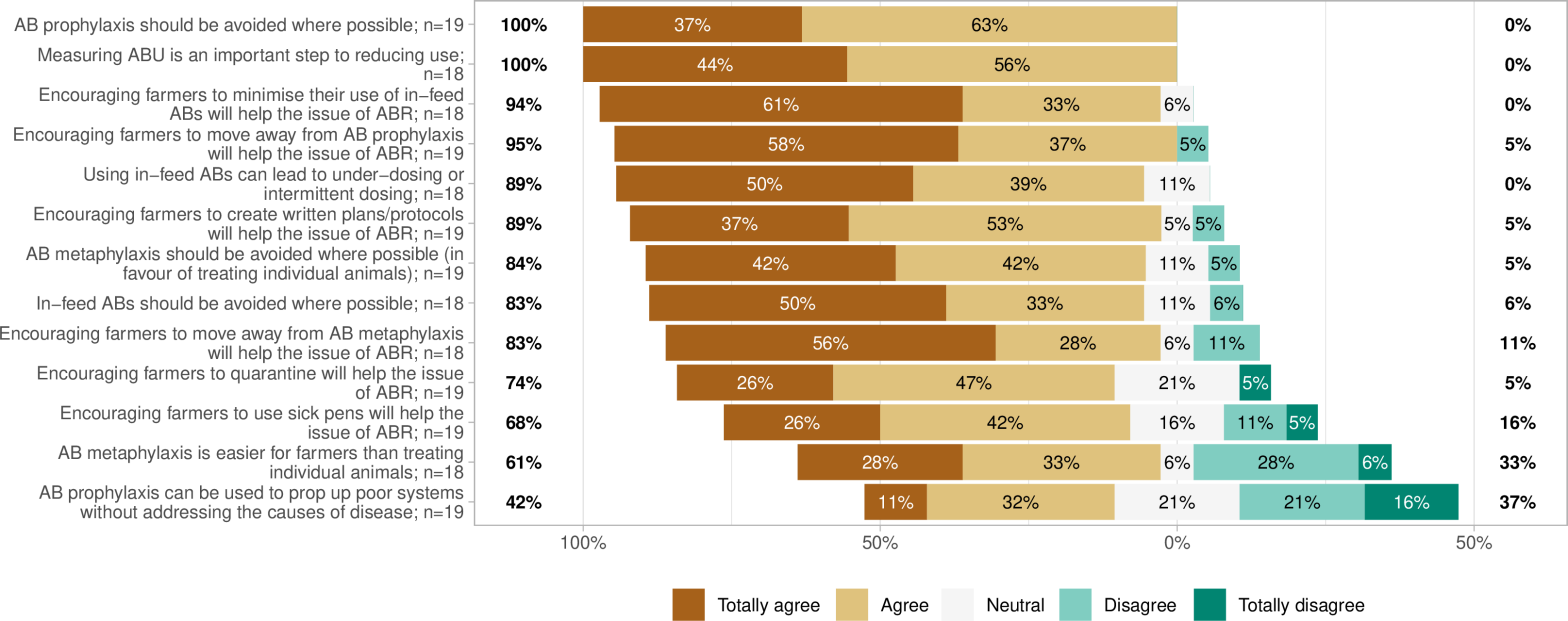
Likert chart showing the response to 13 statements regarding the effect of treatment and management approaches to bovine respiratory disease (BRD) on antibiotic resistance (ABR).

To one statement “Antibiotic prophylaxis can be used to “prop up” poor systems without addressing the causes of disease” respondents were split between some level of agreement (42%), neutral (21%) and some level of disagreement (37%; *n* = 8, 4, 7 of 19, respectively; Figure 10).

**Figure 11:**
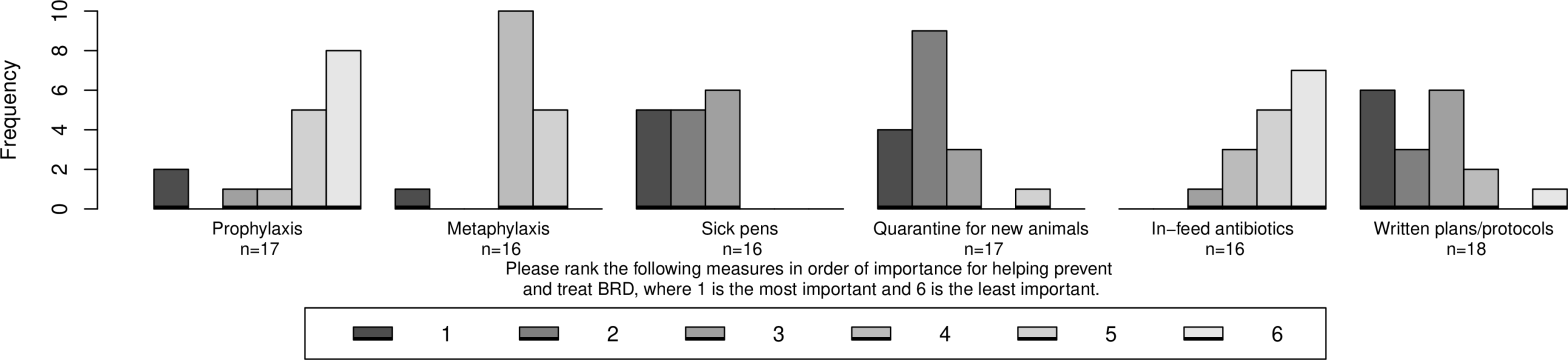
Ranks given to six measures in order of their importance for helping prevent and treat bovine respiratory disease (BRD).

Respondents were asked to rank six measures in order of importance for helping prevent BRD from 1 to 6 with 1 being the most important. The median rank was 2 for “quarantine for new animals” and “sick pens” (IQRs = 0.00 and 2.00, *n* = 17 and 16, respectively); 2.5 for “written plans/protocols” (IQR = 2.00, *n* = 18); 4 for antibiotic metaphylaxis (IQR = 1.00, *n* = 16); and 5 for antibiotic prophylaxis and in-feed antibiotics (IQR = 1.00 and 1.25, *n* = 17 and 16, respectively; Figure 11).

**Figure 12:**
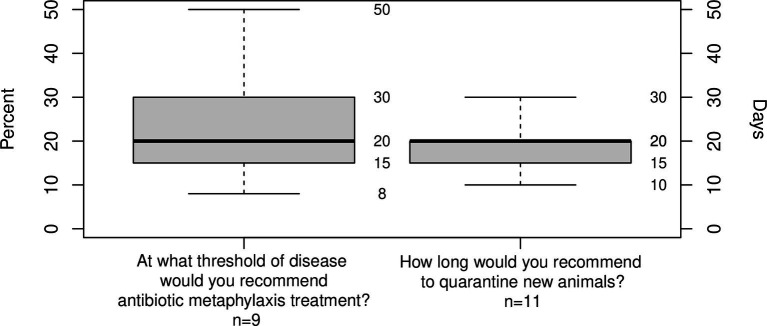
Ranks given to three measures in order of their importance to reducing antibiotic resistance (ABR).

Respondents were also asked to rank three of the measures in terms of their importance to reducing ABR from 1 to 3 with 1 being the most important. The median rank was 2 for “quarantine of new animals” and “sick pens” (IQR = 1.00 and 1.75, *n* = 16 and 18, respectively); and 3 for written plans/protocols (IQR = 2.00, n = 17; Figure 12).

**Figure 13:**
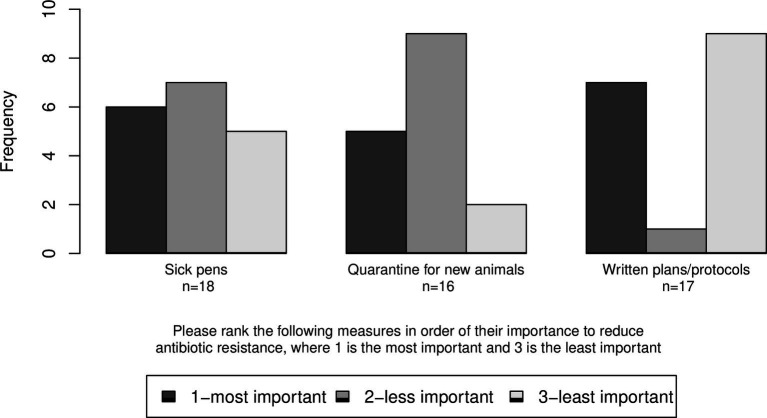
Ranks given to three measures in order of their contribution to the problem of antibiotic resistance (ABR).

Respondents were also asked to rank three of the measures in terms of their contribution to the problem of ABR from 1 to 3 with 1 being the highest contributor. The median rank was 1 for “antibiotic prophylaxis” (IQR = 1.00, *n* = 18); 2 for “in-feed antibiotics” (IQR = 2.00, *n* = 16); and 3 for “antibiotic metaphylaxis” (IQR = 1.00, *n* = 17; Figure 13).

**Figure 14:**
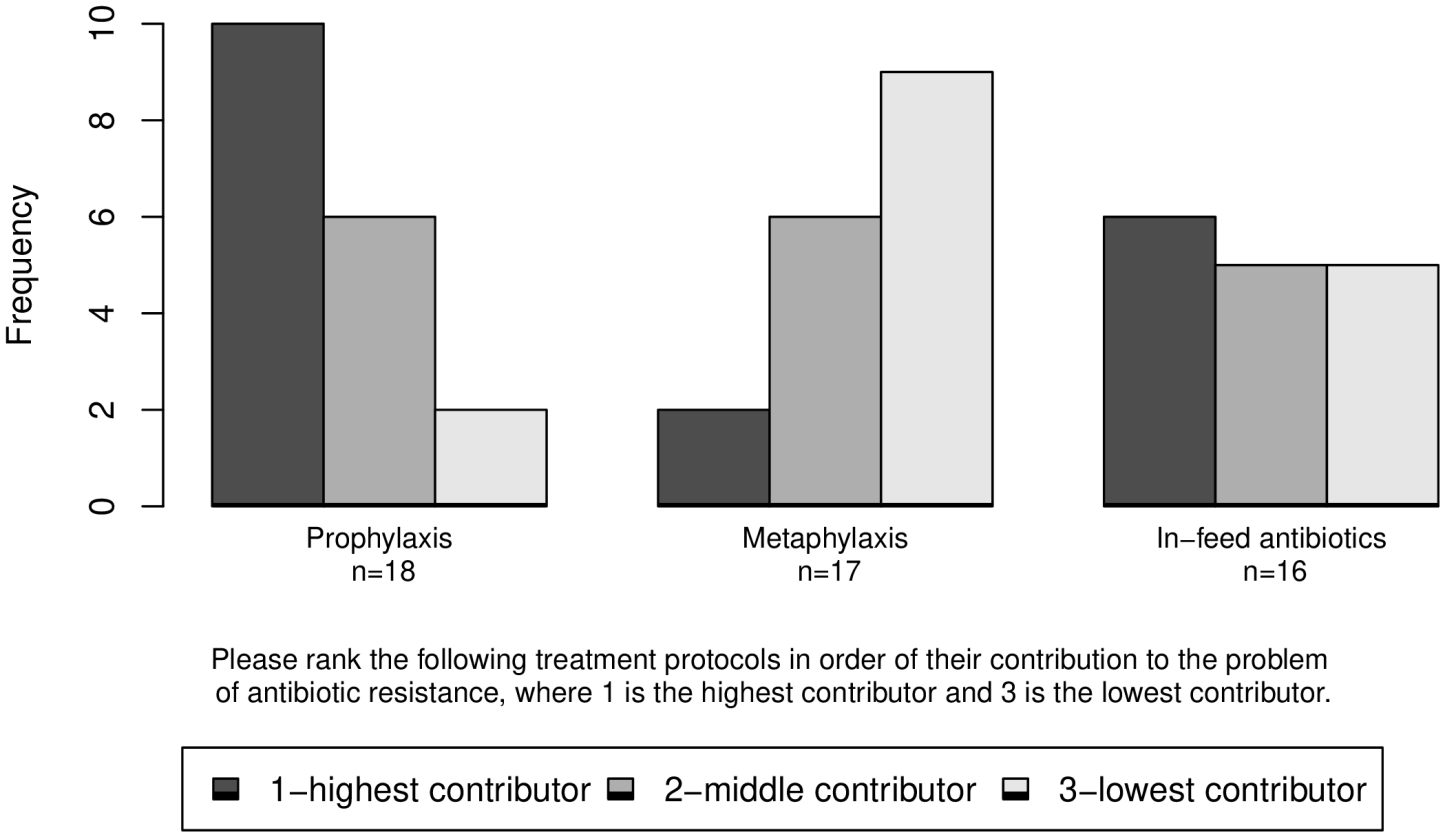
Box plots showing (left) disease level in a group to recommend antibiotic metaphylaxis (%); (right) recommended duration of quarantine for new animals (days). Whiskers show range, box shows lower quartile, median (bold) and upper quartile.

Respondents were asked at what threshold of disease they would recommend antibiotic metaphylaxis, the median answer was 20% (IQR = 15%, *n* = 9). They were also asked how long they would recommend quarantine for new animals; the median answer was 20 days (IQR = 5 days, *n* = 11; Figure 14).

### 3.4 Attitudes on antibiotic use

Main themes and subthemes were collated (Table 1).

**Table 1:** Main themes, sub themes and data collection methodologies.

Theme	Sub-themes	Data collection methodology		
		ABU survey	KAA survey	Focus Group
Antibiotic use	Group vs. Individual Treatment In-feed Antibiotics Growth Promoters	✓	✓ ✓	✓ ✓
Management factors affecting antibiotic use	Purchasing/Adaptation Vaccination Quarantine Hospital pens Animal Handling	✓	✓ ✓ ✓	✓ ✓
Distal causes of antibiotic use	One Health: Comparative performance and ‘Buy-in’ to the Issue of ABR Knowledge and knowledge transfer Records/Controls		✓ ✓	✓ ✓ ✓
Wider issues affecting antibiotic use	Economic situation Political climate			✓ ✓

One of the major factors influencing antibiotic use on farm was decision making around **group treatments** namely whether to treat prophylactically. Antibiotic prophylaxis was seen by farmers to mitigate the risk of a widespread BRD outbreak. The disease risk of a certain group of animals was thought to be influenced by the season, where the animals were bought from and attitudes to risk were influenced by previous disease outbreaks experienced by the farmer.

*“We give antibiotics to the entire batch twice. The first 40–50 days you have pneumonia unless you catch it quickly. The biggest problems are in April–May–June. Last year we gave everyone tilmicosin and also in their food, and only had one case of pneumonia.”* ABU survey, Farmer 13.

Metaphylaxis was thought of as a tool to be used promptly at the first sign of disease in a group to reduce the disease outbreak; the convenience of treating a whole group was seen as an advantage to the farm management.

*“I believe that metaphylaxis in complete pens is justifiable when a large percentage of the total number of animals in the pen is affected.”* KAA survey, Vet 12.

There was a sense of a growing awareness, especially by vets, that prophylactic antibiotic treatment was often used to “prop up” poor farming practises and while effective did not get to the root cause of the disease challenge on that particular farm. This risk of antibiotic resistance meaning that future treatments may not be successful was also a concern.

*“Antibiotic prophylaxis should not be used ... If there are deficient systems, the issue is to find that deficiency, recognize it and try to solve it, but not with the indiscriminate use of antibiotics.”* KAA survey, Vet 6.

The focus group participants held the belief that other regions used significantly more antibiotics than their region, the reasons for this higher use were thought to be greater risk of BRD and different, larger-scale farming businesses.

*“Nobody here uses antibiotics as a preventative.”* Focus Group, Farmer 4.

**In feed antibiotics** were seen by vets to be an inefficient way of treating a clinically sick animal due to the sick animal’s lower desire to eat. There were also concerns over managing the dose rate to prevent underdosing and overdosing, both of which were seen to be problematic with underdosing linked to the idea of AMR.

*“It is very important to keep in mind that for the antibiotic in the diet to work, the animal has to eat and generally the sickest ones do not eat. So, there is a dosage for some and an overdose in others. It is not recommended.”* KAA survey, Vet 11.

**Monensin** (given in the feed) was seen as a useful feed additive that was believed to improve feed-conversion efficiency; its status as an antibiotic was widely debated and, in general, farmers did not consider it to be classified as an antibiotic or to see harm in using monensin.

*“No, we did not use antibiotics, except for monensin…That’s why it helps you... the help that monensin gives you.”* Focus Group, Farmer 3.

### 3.5 Attitudes on management factors affecting antibiotic use

The decisions around the **purchasing** of new animals (predominately weaned calves) were heavily influenced by previous negative experiences of purchasing from certain farms or regions. The ability of weaned calves to adapt quickly to the feedlot system was an important factor when considering from where to purchase animals (and affected decisions around antibiotic use). It was understood that calves experience significant upheaval when they are weaned, transported and have to adapt to a new diet within the space of a few days.

*“Those we bring from Buenos Aires we treat with tilmicosin. We use tilmicosin as if it were water ... The animals that come from Buenos Aires, because it is a farm that comes with much more movement. You notice that the first days are more prone to pneumonia. Considering that these animals spend three days at the market and then the journey, a touch of antibiotic does not hurt.”* ABU Survey, Farmer 12.

**Vaccination** was seen to be important for lowering disease risk, including the risk of BRD, and as such was associated with lower antibiotic usage. Some participants were concerned about the ability of stressed calves to immunologically respond to vaccines when given at the grower/finisher farm as opposed to the breeding farm of origin.

*“Use vaccines to prevent disease and avoid the use of antibiotics.”* KAA survey, Vet 13.

**Quarantine** of new animals was seen as a useful tool by some to reduce the risk of introducing disease and antibiotic-resistant bacteria but a practical challenge by others due to the current setup of their pens.

*“The spread of antibiotic resistant BRD (from the previous farm) can be prevented.”* KAA survey, Vet 12.

**Hospital pens** were similarly seen as helpful for treating and managing individual animals but the benefits of hospital pens especially with regard to ABR were not seen by all.

*“In hospital pens, [treatment] can be more individualised and thus avoid doing the same treatment, thus avoiding resistance.”* KAA survey, Vet 11.

Improvements in **animals handling** techniques and facilities had been observed over the last few years and this was seen to reduce stress in the animals being handled and foster a positive relationship between handlers and animals which had long term benefits. The adopting of new farming techniques including rotational mob grazing further aided this relationship between handler and animals.

*“If the pasture in this field is nearly finished and we open the gate, they go by themselves because they already know that they are going to a pasture with more grass.”* Focus Group, Farmer 5.

### 3.6 Attitudes on distal causes of antibiotic use

Focus group participants understood, in general, **the role of farmers** with regard to antibiotic stewardship and were aware of **the challenge of AMR** within human medicine however they considered themselves to be low users of antibiotics compared to other farmers in other regions. They were aware that Latin America and specifically Argentina had lower controls on the sale and use of antibiotics in both human and veterinary medicine compared to systems in place in Europe and North America, but they felt that these systems were not infallible.

*“You were talking about the animals, the producers. But a pharmacy gives you an antibiotic and you do not even need a prescription, nothing.”* Focus Group, Farmer 1.

*“Yes, in general, the situation of general control here is general in Latin America, it is terrible, terrible.”* Focus Group, Vet 1.

*“In that sense, there is not much social awareness, is there?”* Focus Group, Farmer 2.

Vets considered that their role as veterinarians included **educating** and informing farmers of the risks of AMR and felt that farmers should use their knowledge to make informed treatment decisions and create treatment plans and protocols.

*“A strong campaign must be carried out on the responsible use of antibiotics, aimed at professionals and producers.”* KAA survey, Vet 2.

The focus group showed some confusion over what constituted an antibiotic, how antibiotic resistance develops and how this may affect humans though they showed a strong awareness of anti-parasitic resistance.

*“But how long does it take for resistance to develop? How much time do you have to put in for an animal to become resistant because I assumed that most of us buy calves and sell steers, we buy weaners and sell them for rearing?”* Focus Group, Farmer 1.

Vets considered that the use of on-farm medicine **records** was useful for combatting AMR and that greater traceability within the supply chain was needed. The focus group mentioned the use of laboratory diagnostic support to tailor antibiotic treatments but mentioned that the time in processing samples was too long to be useful. The view on regulator **control** was broadly negative with the main organisation SENASA (The Argentine National Food Safety and Quality Service) thought to be more interested in paperwork and tax collection than animal welfare or food safety.

*“That control is actually towards practically purely external and tax issues and not health issues. There is not even a SENASA control here.”* Focus Group, Farmer 2.

### 3.7 Attitudes on wider issues affecting antibiotic use

The focus group discussed the **economic situation** in Argentina and the challenges this brought to farming. The main challenges centred around the high rate of inflation and the government control of the price of beef which was affecting the way the farmers and vets operated. Large changes in the beef price at certain times of the year and the rate of inflation meant that farmers would sell fat animals individually instead of in bulk as any savings lost value in the bank. Decision making around whether to fatten animals in feedlots or on pasture were strongly affected by the price and predictability of feedstuffs with the cheapest, safest option to fatten animals more slowly on grass. Investing in farm businesses was seen to be difficult and this prevented farmers from adopting new technologies.

*“That is the ‘pocket money’ day because they are selling little by little, they cannot sell everything. What do they do with the money?”* Focus Group, Vet 1.

The focus group also identified problems with the **political climate** at the time including cronyism, bureaucracy and corruption. This led to the feeling that there were many people employed in public office that requested large amount of paperwork from farmers but knew little about farming and had little sympathy with farmers.

*“We here, in reality in Argentina, we have a quite important problem that public institutions generally are a cave of mediocrity, it should not happen that people who are friends of the government end up being incorporated into institutions like SENASA without any knowledge.”* Focus Group, Farmer 3.

## 4 Discussion

The first aim of the project was to understand how antibiotics are currently used across the beef sector in Argentina. The quantitative analysis demonstrated a similar pattern of antibiotic use as that seen in feedlot systems in North America, with ubiquitous use of monensin along with a limited range of other antibiotic classes for the control of respiratory and ocular infections in particular. Over the past several decades Argentina has adopted and adapted many of the practices of large scale, intensive and semi-intensive ‘feedlot’ cattle finishing, first developed in North America (35–37). Specific patterns of ABU are associated with these feedlot systems as they provide a means of treating or preventing bacterial infections which are more prevalent under these husbandry conditions such as bovine respiratory disease (BRD) (38, 39). ABU can be broadly segmented by administration route into either in-feed/oral antibiosis of groups vs. individual treatment of individuals by injection. When we seek to compare patterns of usage between countries, we need to first consider the regulatory environment in which these farms operate. Regulation on the permitted antibiotic classes and route of administration have a profound impact upon patterns of ABU. In-feed antibiotics, and monensin in particular, are good examples of this regulatory diversity. Monensin has been used extensively throughout the world in cattle finishing systems and other livestock species since the 1970’s and is not considered to be a medically important antibiotic in some jurisdictions and therefore regulated as a feed additive rather than as an antibiotic (40). In contrast, the United Kingdom, EU, and others consider it an antibiotic on the basis of its mechanism of actions and regulate it as such. Its use is heavily controlled and regulated under the same antibiotic framework as other classes used in both veterinary and human medicine (41). Other antibiotic classes, principally tetracyclines and macrolides, are also administered as in-feed preparations with few restrictions in countries such as the United States while the use of these classes by this route is heavily restricted or prohibited in regions such as the EU and United Kingdom, while their administration as an injectable preparation is widely accepted and regulated on a broadly similar basis globally including South America (42, 43). This substantial structural difference in antibiotic availability means that when we compare antibiotic usage between countries, we must define the type of usage in order to ensure comparisons are meaningful, useful, and fair.

Very few quantitative estimates of antibiotic usage in beef cattle production systems have been published and the lack of standardised methods for data collection or calculation of denominator metrics hamper accurate comparisons between countries. In the United Kingdom and EU mixed species farming systems are far more common than exclusively beef cattle systems and this factor must be accounted for when attempting to estimate farm level ABU. For example, Humphry et al. (44) attempted to estimate beef antibiotic usage but failed to adequately account for the presence of sheep in their study design and thus introduced a very significant potential bias into their usage estimates leading to the likely overestimation of antibiotic usage in beef cattle. However, a small number of studies have robustly estimated ABU from sale/purchase data using broadly similar recruitment strategies and inclusion criteria which reduce the potential bias that may arise from structural differences in farm business structure and demography (10, 26, 45, 46). These four studies were of a comparable size and similar time periods to the current study and thus provide a useful, if imperfect, comparison between beef production systems between three continents. Collecting antibiotic data via sales records has been shown to be a suitable way to collect ABU data from farms (47) and extensively used for comparative studies between farm types and livestock sectors (29, 48–50). In Argentina, this process is complicated by prescription-free antibiotic purchases and the range of potential vendors.

When we compare the usage of non-monensin antibiotic classes a similar reliance upon tetracyclines and macrolides was observed in the sample of Argentine farms and North American farms while a wider range of antibiotics were used in UK beef cattle. However, while the antibiotic class preference was similar the route of administration varied profoundly. US and Canadian beef producers (10, 45) reported using far greater quantities of these two classes as oral preparations than the Argentine farms in this study or the UK farms (29). Total medical ABU, which excludes monensin, has been reported to be significantly and substantially higher in US beef feedlots compared to both Argentine and UK beef finishing systems, ranging from 21.01 mg/Kg to 38.93 mg/Kg in the US herds compared to 2.29 mg/Kg in Argentine herds in this study, and 8.02 mg/kg PCU (ESVAC) UK herds (29, 46). In this study we found that group medication rather than individual animal treatments constituted the majority of therapeutic treatments (72.92%); the majority of farms (56%) reported using antibiotic metaphylaxis either occasionally or regularly and routine antibiotic prophylaxis was used in 39% of the farms surveyed. There was not a significant correlation between farm level annual ABU and behavioural risk factors associated with decisions involving prophylaxis and metaphylaxis. However, this may be a consequence of the small sample size relative to the variation in antibiotic usage between farms as opposed to the presence or absence of an underlying relationship between the dependent and independent variables.

Monensin usage was not recorded in a comparable way in the US studies and is largely prohibited in the United Kingdom and EU. Comparisons are therefore difficult to make or interpret. In this study monensin represented more than 94% of total antibiotic usage by mass and its use was ubiquitous among the feedlots in Argentina but accurate quantification of use is challenging due to the manner in which it is incorporated into feed by farmers or feed processors making data collation more difficult than for the other antibiotic types. The relative importance of monensin and other oral antibiotics to the development of AMR compared to other classes is as yet unclear and requires further study in order to allow a more informed cost–benefit analysis of these patterns of use to be appraised. However, the results from this study would suggest that Argentine feedlots have developed antibiosis management practices that more closely resemble North American feedlots than European beef production systems in terms of the pattern of antibiotics used while the quantities used appear to be substantially less than the equivalent feedlots systems in North America. However, the authors emphasise the need for further direct comparative research work, using standardised methods, across international production systems to validate this initial, tentative, interpretation.

The second aim was to identify the factors that currently drive usage. One of the key factors in the use pattern of antibiotics in the Argentina beef sector stems from decisions surrounding purchasing animals and the adaptation period for new animals entering a farm/feedlot. Two thirds of farms purchased calves, and all farms identified the adaptation period as the period with the highest morbidity/mortality. Nine farms sourced at least some of their new stock from farms of unknown health status. Animals travelled up to 900 km to reach the farm and price was cited as the most popular reason when making purchasing decisions. Numerous studies highlight the physiological and epidemiological changes caused by transport stress and adaptation challenges leading to drop a in immune function and increased risk of BRD (51–54). Furthermore, a systematic review and meta-analysis of the effect of group treatments on BRD suggested that use of group treatments could be traced back to issues with the “segmented infrastructure” of the feedlot sector (55).

The third aim was to identify the main drivers of this usage in terms of farmer and veterinary knowledge and attitudes. This paper does not attempt to compare veterinary and farmer attitudes as there is considerable overlap between the two roles. The majority of veterinarians surveyed disagreed that group treatments were the best way to tackle BRD and linked reducing these practices to reducing the risk of ABR. However, the qualitative data analysed highlighted the importance of group treatment for managing or preventing severe outbreaks. In-feed antibiotics for BRD were widely considered by veterinarians surveyed to be a sub-optimal route of administration due to the lack of appetite in sick animals. Baptiste and Kyvsgaard (55) found group treatments “represent major antimicrobial consumption for highly variable short-term gains in absolute risk reduction of morbidity/mortality”; they cautioned against blanket treatments in light of the risks of ABR. Although Dennis et al. (56) highlight the economic value of metaphylaxis to the beef industry.

Vaccination regimes were seen as an effective way to reduce morbidity and therefore the need for antibiotics and one survey respondent suggested encouraging vaccination as part of preconditioning on the breeder farm before the high stress event of weaning/moving. Smith’s (57) review on risk factors for BRD addresses the practical and motivational challenges with improving vaccination prior to weaning and suggests further research into the optimum vaccination protocol. The use of quarantine pens for new animals was seen to have advantages especially when used in conjunction to a vaccination protocol however the practical feasibility of this was questioned. Similarly, the use of hospital pens for sick animals was seen to help prevent the spread of disease but did not necessarily equate to lower ABU. Various studies recommend good hygiene practices such as the use of quarantine and hospital pens as a way to reduce ABU without compromising animal health (58–60). However, these studies often have a European bias, and further research is needed into the practical application of such measures within the farming systems in Argentina.

With regard to education and knowledge levels farmers were often well informed about antiparasitic resistance and emerging farming methods such as rotational grazing, however specific knowledge on antibiotics and resistance was often lacking. Farmers were often seen to look to their veterinarian for advice with many larger farms employing a veterinarian full-time. Veterinarians were largely aware of their role in knowledge transfer with one surveyed veterinarian encouraging further methods of communication to aid knowledge transfer from veterinarians to farmers. Vet-farmer relationships are important for knowledge transfer including within the topic ABR; Bokma et al. (61) conducted a review of observation studies that showed that poor relationships between veterinarians and farmers and poor farmer knowledge were linked to higher ABU.

Many participants in our project saw themselves as low users but were aware of others that they saw as higher users; some of the low reported use could be attributed to the Dunning–Kruger effect which states that the majority of people tend to report better than average performance (62). Comparisons were made to other parts of the world namely Europe and United States which were seen as more heavily controlled in terms of regulation surrounding antibiotics and to other sectors namely human medicine in Argentina which was seen to be contributing to the problem of ABR. Phrases such as “responsibility,” “social awareness” and “social conscience” were used in relation to farmers’ position in society. The phenomenon of othering is common across many sectors; a survey of professionals involved with ABU across all sectors in Canada found that “across participant responses to multiple questions, there was emergence of 2 cross-cutting themes: (1) a One Health understanding of antimicrobial stewardship, and (2) blame placed on others for the lack of antimicrobial stewardship success” (63).

Survey participants saw written plans/protocols and the measuring of antibiotic usage on-farm as important parts of antibiotic stewardship. The focus group saw that farmers were open to the idea of changes to the current regulatory system. The FAO (64) recommends farm-level monitoring as a way of reducing ABU following the tenet “you cannot manage what you cannot measure” (65). Studies involving dairy farms in Europe have proven that farm-level health planning can reduce ABU without negatively impacting animal health (66, 67).

It was made clear that the current economic situation in Argentina had had considerable impact on farming practices and on-farm decision making together with a dissatisfaction with the political system due to issues of cronyism, bureaucracy, and corruption. Though the economic situation in Argentina is relatively unique, Iskandar et al.’s (68) review of “drivers of ABR transmission in low-and middle-income countries from a “one health” perspective” stated that the problem of ABR will continue “if governments do not prioritise the “One health” approach and if individual’s accountability is still denied in a world struggling with profound socio-economic problems.” Collecting qualitative data about a sensitive topic can be challenging however the focus group proved productive with farmers and veterinarians feeling comfortable discussing sensitive topics. By choosing participants that knew each other rapport was established quickly however this introduced a level of sampling bias. Although the authors believe that data saturation of the qualitative themes was reached via the different methodologies additional focus groups would have helped to add weight to this belief. The project was limited by the impact of the COVID pandemic preventing in-person fieldwork. Challenges around engagement for focus groups, especially in the more commercial farming areas were significant with farmers seeing others as rivals and we found no existing peer-to-peer groups in the Tandil area. This is a crucial difference compared to European farmers that often engage in peer-to-peer groups either through their vet practice or independently and this approach has been shown to aid in improving antibiotic stewardship (69).

In conclusion, Argentine beef farms that took part in the study resemble North American beef farms in terms of antibiotic practices but with considerably lower usage. However, while this comparison is a useful starting point it should be emphasised that truly representative samples of beef producers antibiotic use are not available from any country at present. All studies, including this one, have been relatively small scale, convenience samples subject to participation bias. Furthermore, the methods for collecting and analysing antibiotic usage differ in both the definitions used for inclusion/exclusion of numerators and denominators as well as the indicator metrics for quantification. There is therefore an obvious and pressing need to address these research gaps with larger scale, multinational studies using harmonised methodologies.

Monensin is widely used in Argentine beef farms and represents a large proportion of total ABU. The adaptation period presents a challenge to animal health, especially from BRD, and antibiotics are used in a range of measures from prophylaxis to individual treatment depending on farm management practices and perceived risk of disease. Knowledge and education levels on the topic of ABU and ABR in farmers and veterinarians could be improved however there was evidence of an awareness of the social responsibility of the beef farming sector with regard to ABU. Further research into internationally comparable measures of ABU and detailed cost breakdowns of practical on farm interventions are needed to aid improved antimicrobial stewardship in countries such as Argentina.

## Data Availability

The original contributions presented in the study are included in the article/Supplementary material, further inquiries can be directed to the corresponding author.
