# Supplementary material for: A mixed method approach to analysing patterns and drivers of antibiotic use and resistance in beef farms in Argentina
Source: Front Vet Sci. 2024 Nov 13;11:1454032. doi: 10.3389/fvets.2024.1454032 (PMC11600977; doi:10.3389/fvets.2024.1454032)
Supplement: Supplementary file 1 [file Data_Sheet_1.zip › Document 6.docx]

TEXTO DE LA ENCUESTA

1: Indique su nivel de acuerdo con las siguientes opiniones sobre el uso de profilaxis antibiótica para ERB (Enfermedad respiratoria bovina)(es decir, el tratamiento de corrales enteros de animales que ingresa con antibióticos como prevención de ERB, sin que ninguno de los animales muestre sí­ntomas).

Niveles: Totalmente de acuerdo, De acuerdo, Neutral, En desacuerdo, Totalmente en desacuerdo

1.1. La profilaxis antibiótica es la forma más eficaz de controlar la ERB

1.2. Se debe evitar la profilaxis antibiótica siempre que sea posible

1.3. Alentar a los productores a reducir la profilaxis con antibióticos ayudará a resolver el problema de la resistencia a los antibióticos

1.4. La profilaxis con antibióticos se puede usar para "apuntalar" sistemas deficientes sin abordar las causas de las enfermedades.

2: ¿Tiene algún otro comentario sobre el uso de profilaxis antibiótica para controlar la ERB (especialmente con respecto al tema de la resistencia a los antibióticos)? [texto libre]

3: Indique su nivel de acuerdo con las siguientes opiniones sobre el uso de metafilaxis antibiótica para ERB (el tratamiento de corrales completos con antibióticos cuando algunos de los animales comienzan a mostrar sí­ntomas de ERB, en lugar de tratar animales individuales).

Niveles: Totalmente de acuerdo, De acuerdo, Neutral, En desacuerdo, Totalmente en desacuerdo

3.1. La metafilaxis antibiótica es una forma más eficaz de controlar la ERB que tratar animales individuales

3.2. Siempre que sea posible se debe evitar la metafilaxis antibiótica (en favor de tratar animales individuales)

3.3. Alentar a los productores a reducir la metafilaxis antibióticas ayudará a resolver el problema de la resistencia a los antibióticos

3.4. La metafilaxis antibiótica es más fácil para los productores que tratar a animales individuales

4: ¿Tiene algún otro comentario sobre el uso de la metafilaxis antibiótica para controlar la ERB (especialmente con respecto al tema de la resistencia a los antibióticos)? [texto libre]

5: ¿A partir de qué porcentaje de animales enfermos en el corral recomendaría el tratamiento de metafilaxis con antibióticos a todo el grupo? (como porcentaje del grupo, por ejemplo, 10%) [texto libre]

6: Indique su nivel de acuerdo con las siguientes opiniones sobre el uso de corrales de animales enfermos/enfermerí­a para animales en tratamiento/recuperación de ERB.

Niveles: Totalmente de acuerdo, De acuerdo, Neutral, En desacuerdo, Totalmente en desacuerdo

6.1. El uso de corrales de animales enfermos ayuda a prevenir la propagación de ERB

6.2. Alentar a los productores a utilizar corrales de animales enfermos ayudará a resolver el problema de la resistencia a los antibióticos

6.3. Los animales en el corral de enfermos se recuperan más rápido que los animales tratados en corrales grupales

6.4. Los corrales de animales enfermos deben estar situados lejos de otros corrales (es decir, sin contacto nariz con nariz) para que sean efectivos

7: ¿Tiene algún otro comentario sobre el uso de corrales de animales enfermos/enfermería para animales en tratamiento/recuperación de ERB (especialmente con respecto al tema de la resistencia a los antibióticos)? [texto libre]

8: Indique su nivel de acuerdo con las siguientes opiniones sobre el uso de la cuarentena para animales que ingresan al establecimiento como forma de controlar la ERB.

Niveles: Totalmente de acuerdo, De acuerdo, Neutral, En desacuerdo, Totalmente en desacuerdo

8.1. Poner en cuarentena animales que ingresan al establecimiento ayuda a prevenir la propagación de ERB

8.2. Alentar a los productores a poner en cuarentena animales ayudará a resolver el problema de la resistencia a los antibióticos

8.3. Los corrales de cuarentena deben estar situados lejos de otros corrales (es decir, sin contacto nariz con nariz) para que sean efectivos

9: ¿Durante cuánto tiempo aconsejaría a sus productores que dejen en cuarentena a los animales que ingresan al establecimiento? [texto libre]

10: ¿Tiene algún otro comentario sobre el uso de cuarentena para animales que ingresan al establecimiento como forma de controlar la ERB (especialmente con respecto al tema de la resistencia a los antibióticos)? [texto libre]

11: Indique su nivel de acuerdo con las siguientes opiniones sobre el uso de antibióticos en la alimentación para el tratamiento o la prevención de la ERB.

Niveles: Totalmente de acuerdo, De acuerdo, Neutral, En desacuerdo, Totalmente en desacuerdo

11.1. Los antibióticos en la alimentación son importantes para el tratamiento o la prevención de la ERB

11.2. Siempre que sea posible, se deben evitar los antibióticos en la alimentación.

11.3. Alentar a los productores a reducir el uso de antibióticos en la alimentación ayudará a resolver el problema de la resistencia a los antibióticos

11.4. El uso de antibióticos en la alimentación puede provocar una dosificación insuficiente o intermitente

12: ¿Tiene algún otro comentario sobre el uso de antibióticos en la alimentación para el tratamiento o la prevención de la ERB (especialmente con respecto a la cuestión de la resistencia a los antibióticos)? [texto libre]

13: Indique su nivel de acuerdo con las siguientes opiniones sobre el uso de planes/protocolos escritos para el tratamiento o prevención de ERB.

Niveles: Totalmente de acuerdo, De acuerdo, Neutral, En desacuerdo, Totalmente en desacuerdo

13.1. Los planes/protocolos escritos ayudan a los establecimientos a mejorar su gestión de ERB

13.2. Alentar a los productores a crear planes/protocolos escritos ayudará a resolver el problema de la resistencia a los antibióticos

13.3. Medir el uso de antibióticos es un paso importante para reducir su uso

14: ¿Tiene algún otro comentario sobre el uso de planes/protocolos escritos para el tratamiento o prevención de la ERB (especialmente con respecto al tema de la resistencia a los antibióticos)? [texto libre]

15: Clasifique las siguientes medidas en orden de importancia para ayudar a prevenir y tratar la ERB, donde 1 es la más importante y 6 es la menos importante.

15.1: Profilaxis (tratamiento grupal sin síntomas)

15.2: Metafilaxis (tratamiento grupal con algunos síntomas que muestran)

15.3: Corrales de animales enfermos

15.4: Cuarentena para nuevos animales

15.5: Antibióticos en la alimentación

15.6: Planes/protocolos escritos

16: Clasifique las siguientes medidas en orden de importancia para reducir la resistencia a los antibióticos, donde 1 es la más importante y 3 es la menos importante.

16.1: Corrales de animales enfermos

16.2: Cuarentena para nuevos animales

16.3: Planes/protocolos escritos

17: Clasifique las siguientes decisiones según su contribución al problema de resistencia a los antibióticos, donde 1 es el factor que mayor contribuye y 3 es el menor.

17.1: Profilaxis (tratamiento grupal sin síntomas)

17.2: Metafilaxis (tratamiento grupal con algunos síntomas que muestran)

17.3: Antibióticos en la alimentación

18: En su opinión, ¿qué podrían hacer los productores de feedlot para ayudar con el problema de la resistencia a los antibióticos? [texto libre]
