# Supplementary material for: A mixed method approach to analysing patterns and drivers of antibiotic use and resistance in beef farms in Argentina
Source: Front Vet Sci. 2024 Nov 13;11:1454032. doi: 10.3389/fvets.2024.1454032 (PMC11600977; doi:10.3389/fvets.2024.1454032)
Supplement: Supplementary file 1 [file Data_Sheet_1.zip › Document 5.docx]

SURVEY TEXT

1: Please indicate your level of agreement with the following opinions regarding the use of antibiotic prophylaxis for BRD (Bovine Respiratory Disease) (i.e., the treatment of a group of animals to prevent disease without the presence of symptoms).

Levels: Totally agree, Agree, Neutral, Disagree, Totally disagree

1.1: Antibiotic prophylaxis is the most effective way to control BRD

1.2: Antibiotic prophylaxis should be avoided where possible

1.3: Encouraging farmers to move away from antibiotic prophylaxis will help the issue of antibiotic resistance

1.4: Antibiotic prophylaxis can be used to "prop up" poor systems without addressing the causes of disease

2: Do you have any other comments on the use of antibiotic prophylaxis to control BRD (especially with regard to the issue of antibiotic resistance)? [free text]

3: Please indicate your level of agreement with the following opinions regarding the use of antibiotic metaphylaxis for BRD (i.e., the treatment of a group of animals with a proportion of the group showing symptoms).

Levels: Totally agree, Agree, Neutral, Disagree, Totally disagree

3.1: Antibiotic metaphylaxis is a more effective way to control BRD that treating individual animals

3.2: Antibiotic metaphylaxis should be avoided where possible (in favour of treating individual animals)

3.3: Encouraging farmers to move away from antibiotic metaphylaxis will help the issue of antibiotic resistance

3.4: Antibiotic metaphylaxis is easier for farmers than treating individual animals

4: Do you have any other comments on the use of antibiotic metaphylaxis to control BRD (especially with regard to the issue of antibiotic resistance)? [free text]

5: At what threshold of disease would you recommend antibiotic metaphylaxis treatment to the whole group as opposed to treating individual animals? (as a percent of the group e.g.10%) [free text]

6: Please indicate your level of agreement with the following opinions regarding the use of sick/hospital pens for animals undergoing treatment/recovery from BRD

Levels: Totally agree, Agree, Neutral, Disagree, Totally disagree

6.1: The use of sick pens helps prevent the spread of BRD

6.2: Encouraging farmers to use sick pens will help the issue of antibiotic resistance

6.3: Animals in the sick pen recover faster than animals treated in group pens

6.4: Sick pens need to be situated away from other pens (i.e. no nose-to-nose contact) to be effective

7: Do you have any other comments on the use of sick/hospital pens for animals undergoing treatment/recovery from BRD (especially with regard to the issue of antibiotic resistance)? [free text]

8: Please indicate your level of agreement with the following opinions regarding the use of quarantine for new animals as a way of controlling BRD

Levels: Totally agree, Agree, Neutral, Disagree, Totally disagree

8.1: Quarantining new animals helps prevent the spread of BRD

8.2: Encouraging farmers to quarantine will help the issue of antibiotic resistance

8.3: Quarantine pens need to be situated away from other pens (i.e. no nose-to-nose contact) to be effective

9: How long would you advise your farmers to quarantine new animals for? [free text]

10: Do you have any other comments on the use of quarantine for new animals as a way of controlling BRD (especially with regard to the issue of antibiotic resistance)? [free text]

11: Please indicate your level of agreement with the following opinions regarding the use of in-feed antibiotics for the treatment or prevention of BRD

Levels: Totally agree, Agree, Neutral, Disagree, Totally disagree

11.1: In-feed antibiotics are important for the treatment or prevention of BRD

11.2: In-feed antibiotics should be avoided where possible

11.3: Encouraging farmers to minimise their use of in-feed antibiotics will help the issue of antibiotic resistance

11.4: Using in-feed antibiotics can lead to under-dosing or intermittent dosing

12: Do you have any other comments on the use of in-feed antibiotics for the treatment or prevention of BRD (especially with regard to the issue of antibiotic resistance)? [free text]

13: Please indicate your level of agreement with the following opinions regarding the use of written plans/protocols for the treatment or prevention of BRD

Levels: Totally agree, Agree, Neutral, Disagree, Totally disagree

13.1: Written plans/protocols help farms to improve their management of BRD

13.2: Encouraging farmers to create written plans/protocols will help the issue of antibiotic resistance

13.3: Measuring antibiotic use is an important step to reducing use

14: Do you have any other comments on the use of written plans/protocols for the treatment or prevention of BRD (especially with regard to the issue of antibiotic resistance)? [free text]

15: Please rank the following measures in order of importance in helping to prevent and treat BRD, where 1 is the most important and 6 is the least important.

15.1: Prophylaxis (group treatment without symptoms)

15.2: Metaphylaxis (group treatment with some showing symptoms)

15.3: Sick pens

15.4: Quarantine for new animals

15.5: In-feed antibiotics

15.6: Written plans/protocols

16: Rank the following measures in order of importance to reduce antibiotic resistance, where 1 is the most important and 3 is the least important.

16.1: Sick pens

16.2: Quarantine for new animals

16.3: Written plans/protocols

17: Rank the following treatment protocols in order of their contribution to the problem of antibiotic resistance, where 1 is the largest contributor and 3 is the smallest.

17.1: Prophylaxis (group treatment without symptoms)

17.2: Metaphylaxis (group treatment with some showing symptoms)

17.3: In-feed antibiotics

18: In your opinion what is the single best thing feedlot farmers can do to help with the problem of antibiotic resistance? [free text]
