# Supplementary material for: A mixed method approach to analysing patterns and drivers of antibiotic use and resistance in beef farms in Argentina
Source: Front Vet Sci. 2024 Nov 13;11:1454032. doi: 10.3389/fvets.2024.1454032 (PMC11600977; doi:10.3389/fvets.2024.1454032)
Supplement: Supplementary file 1 [file Data_Sheet_1.zip › Document 4.docx]

Guía de discusión de grupo de enfoque

Gracias por tomarse el tiempo de ser parte de este grupo de enfoque. Este es un grupo de enfoque sobre el tema de la resistencia a los antimicrobianos (RAM).

*Estimamos un tiempo de 10 minutos por sección.*

1. **Conocimiento y difusión:** ¿Se sienten conocedor de RAM? ¿De dónde reciben su información sobre RAM? ¿Has visto ejemplos de (casos) RAM personalmente? ¿Hablan sobre RAM con sus veterinarios? ¿Reciben información sobre RAM de otros proveedores de antibióticos (como los laboratorios/farmacias)?
2. **Importancia:** ¿Qué importancia cree que tiene el tema de la RAM en general? ¿Cree que la RAM debería tener una prioridad mayor o menor de la que tiene actualmente?
3. **Rol**: ¿Cuál es su rol como productores con respecto a la resistencia a los antibióticos? ¿Qué pasa con otros sectores que utilizan antibióticos, por ejemplo, hospitales, otros tipos de establecimiento y otros países?
4. *[pausa]*
5. **Principales impulsores:** ¿Cuáles son los principales impulsores de la RAM? ¿Cuáles son las principales razones para el uso de antibióticos en sus campos? ¿Creen que reducir el uso de antibióticos es práctico?
6. **Enfermedad respiratoria bovina:** En relación con la enfermedad respiratoria bovina, ¿cuáles son las mejores formas de reducir el uso de antibióticos sin afectar negativamente el bienestar animal? ¿Qué sucede con el pre-acondicionamiento? ¿Cuarentena? ¿El uso de corrales para animales enfermos, la profilaxis y la metafilaxis?
7. **Cambios más generales**: ¿Qué cambios más generales serían los más útiles para reducir el uso de antibióticos en los campos?

Muchas gracias por su tiempo y apoyo con este proyecto.
