# Supplementary material for: A mixed method approach to analysing patterns and drivers of antibiotic use and resistance in beef farms in Argentina
Source: Front Vet Sci. 2024 Nov 13;11:1454032. doi: 10.3389/fvets.2024.1454032 (PMC11600977; doi:10.3389/fvets.2024.1454032)
Supplement: Supplementary file 1 [file Data_Sheet_1.zip › Document 3.docx]

Focus Group Discussion Guide

Thank you for taking the time to be part of this focus group. This is a focus group on the topic of antimicrobial resistance (AMR).

*We estimate a time of 10 minutes per section.*

1. Knowledge and dissemination: Do you feel knowledgeable about AMR? Where do you get your information on AMR? Have you personally seen examples of AMR (cases)? Do you talk about AMR with your vet? Do you receive information on AMR from other antibiotic suppliers (such as laboratories/pharmacies)?
2. Importance: How important do you think the issue of AMR is in general? Do you think AMR should have a higher or lower priority than it currently has?
3. Role: What is your role as farmers with regard to antibiotic resistance? What about other sectors that use antibiotics, e.g. hospitals, other types of farms, and other countries?
4. *[pause]*
5. Main drivers: What are the main drivers of AMR? What are the main reasons for antibiotic use on your farms? Do you think reducing antibiotic use is practical?
6. Bovine respiratory disease: Regarding bovine respiratory disease, what are the best ways to reduce antibiotic use without negatively impacting animal welfare? What about preconditioning? Quarantine? Use of sick pens, prophylaxis, and metaphylaxis?
7. More general changes: What more general changes would be most helpful in reducing antibiotic use on farms?

Thank you very much for your time and support with this project.
