# Supplementary material for: A mixed method approach to analysing patterns and drivers of antibiotic use and resistance in beef farms in Argentina
Source: Front Vet Sci. 2024 Nov 13;11:1454032. doi: 10.3389/fvets.2024.1454032 (PMC11600977; doi:10.3389/fvets.2024.1454032)
Supplement: Supplementary file 1 [file Data_Sheet_1.zip › Document 2.docx]

**“Mapeo de patrones, uso y resistencia de antibióticos en la industria cárnica bovina Argentina”**

*NOTA: La información suministrada es confidencial y anónima. La misma será utilizada EXCLUSIVAMENTE con fines académicos y de investigación y los resultados obtenidos del trabajo serán oportunamente informados a los colaboradores de la presente encuesta.*

**Contacto:** Dra. Maria Laura Galotta [galottalaura@vet.unicen.edu.ar](mailto:galottalaura@vet.unicen.edu.ar)

| Establecimiento ID____________________________________________________ |
| --- |
| Rol del entrevistado: __________________________________________________ |
| Periodo **marzo 2019- marzo 2020** |

| **INFORMACION GENERAL** |
| --- |

| 1. Número de animales que ingresaron durante marzo 2019- marzo 2020 |  |
| --- | --- |

| 1. Número de animales vendidos (o faenados) marzo 2019- marzo 2020 |  |
| --- | --- |

| 1. Promedio (Kg) de los animales al inicio |  |
| --- | --- |

| 1. Promedio (Kg) de los animales de terminación |  |
| --- | --- |

| 1. Tipo de engorde (Seleccione 1 o más opciones, e indique en porcentaje según corresponda) |
| --- |

| Hotelería o profesional |  |
| --- | --- |
| Engorde a corral casero |  |
| Destete precoz a corral |  |
| Pastoril con suplementación |  |

| 1. Origen y número de los animales que engordan (Seleccionar 1 o más opciones, e indique en porcentaje según corresponda) |
| --- |

| Feria |  |
| --- | --- |
| Campo con sanidad conocida (Propio) |  |
| Campo con sanidad conocida (Tercero) |  |
| Campo sanidad desconocida |  |
| Desconocido |  |

| 1. Categoría que se engordan en el Feedlot (Especificar número de animales por ciclo): |
| --- |

| **Categoría** | **Número/Observaciones** | **Duración del ciclo** (ej. Terminación del animal peso/ días/ otro) |
| --- | --- | --- |
| Ternero/a destete precoz |  |  |
| Ternero/a destete convencional |  |  |
| Novillitos 1-2 años |  |  |
| Novillito 2-3 años |  |  |
| Vaquillona 1-2 años |  |  |
| Vaquillona 2-3 años |  |  |
| Vaca |  |  |
| Toro |  |  |
| MEJ (Macho Entero Joven) |  |  |

| 1. ¿Cuáles son las razas presentes en el establecimiento? | |
| --- | --- |
|  | - Aberdeen Angus |
|  | - Hereford |
|  | - Braford |
|  | - Brangus |
|  | - Criollo |
|  | - Holando Argentino |
|  | - Otras |

| 1. Mencione el proceso de compra de animales:  - ¿De qué región (provincias) de la Argentina vienen?   __________________________________________________________________  __________________________________________________________________   - ¿Qué situación lo hace cambiar el origen de los animales?   __________________________________________________________________  __________________________________________________________________   - ¿Cuál es la distancia promedio que viajan los animales para ingresar al establecimiento?   _________________________________________________________________   - ¿Usted observa mayor número de animales enfermos dependiendo el origen de los animales? Si la respuesta es sí, ¿de qué región?   __________________________________________________________________  __________________________________________________________________ |
| --- |

1. ¿Cuál es el destino de los animales (ejemplo: consumo interno o exportación)?

__________________________________________________________________

| **INSTALACIONES** |
| --- |

| 1. Cantidad total de corrales |  |
| --- | --- |
| 1. Tamaño de los corrales   __________________________________________________________________ | |
| 1. Tipo de suelo (material) donde están ubicados los corrales  \| Tierra \|  \| Concreto \|  \| Mixto \|  \| Otros \|  \| \| --- \| --- \| --- \| --- \| --- \| --- \| --- \| --- \| | |
| 1. ¿Presencia de pendientes en el establecimiento? (Si la respuesta es Si, indicar donde)   __________________________________________________________________ | |
| 1. ¿Me podría decir como es la distribución de los animales en los corrales? (Carga animal por corral)   __________________________________________________________________  __________________________________________________________________ | |
| 1. ¿Cuál es el criterio para armar los grupos de animales por corral? (ej: Edad, peso, machos/hembras, establecimiento de origen)   __________________________________________________________________ | |
| 1. ¿Se modifican los grupos de animales durante el período de engorde? (por ejemplo, ¿animales de crecimiento lento en un grupo se cambian a un grupo posterior de ganado más joven?)   __________________________________________________________________  __________________________________________________________________ | |
| 1. ¿Hay un corral de enfermería? Si la respuesta es sí, indique cuantos animales hay actualmente en ese corral.   __________________________________________________________________  __________________________________________________________________ | |
| 1. ¿Qué criterio es usado cuando decide mover los animales a un corral de enfermería?   __________________________________________________________________ | |
| 1. Los animales en este corral de enfermería, una vez recuperados, ¿retoman a su corral original, se dirigen a otro corral o permanecen en la enfermería hasta que son enviados a faena o mueren?   __________________________________________________________________  __________________________________________________________________ | |
| 1. ¿Son las calles de circulación y alimentación las mismas? ¿Usted siente que esto le general algún inconveniente en su rutina?   __________________________________________________________________ | |
| 1. ¿Como están ubicadas las bebidas? (Dato importante comparten bebida los corrales)   __________________________________________________________________ | |
| 1. ¿Hay presencia de cortinas forestales?   __________________________________________________________________ | |
| 1. ¿Cómo son los comederos? (material, diseño, plataforma)   __________________________________________________________________ | |
| 1. ¿Hay presencia de sombra en los corrales? (material, localización, etc)   __________________________________________________________________ | |
| \| Si \|  \| No \|  \| \| --- \| --- \| --- \| --- \|  1. ¿Usted realiza algún manejo integrado de plagas?   Si la respuesta es Si, mencione el manejo que realiza:  __________________________________________________________________ | |
| 1. Me podría indicar la fuente de agua de bebida de los animales (ej. agua superficial, agua subterránea). Si es de agua subterránea, ¿cuál es la profundidad de la perforación?   __________________________________________________________________  __________________________________________________________________ | |
| **MANEJO DE LOS ANIMALES** | |

| 1. ¿Cuál es la forma de identificación de los animales? (caravana, caravana electrónica, tatuaje, etc)   __________________________________________________________________ | | | | | |
| --- | --- | --- | --- | --- | --- |
| 1. ¿Cuándo realiza el pesaje de los animales (ej: al ingreso, luego de 4 semanas)   __________________________________________________________________ | | | | | |
| 1. ¿Cuántas veces son pesados los animales hasta su venta/faena?   __________________________________________________________________ | | | | | |
| 1. ¿Utiliza corral de cuarentena al ingreso de los animales? | | | | | |
| Si |  | No |  |  |  |

Si la respuesta es Si, indique por cuanto tiempo

___________ días

| 1. **Sanidad al ingreso de los animales**  - ¿Realiza algún manejo para disminuir el estrés? Si la respuesta es Si, indique cual es el manejo.   __________________________________________________________________   - ¿Usted realiza algún manejo sanitario al ingreso de los animales? Si la respuesta es Si, indique cuando lo realiza (apenas ingresan los animales, después de dos días)   __________________________________________________________________ |
| --- |

Complete según corresponda la sanidad que realiza al ingreso:

| \| Si \|  \| No \|  \| \| --- \| --- \| --- \| --- \|  1. Antiparasitario   Principio activo ____________ Marca: __________ Dosis:______ Lapso de tiempo________ |
| --- | --- | --- | --- | --- |
| \| Si \|  \| No \|  \| \| --- \| --- \| --- \| --- \|  1. Vacunas enfermedades respiratorias     Marca:__________________ Dosis:____________ Lapso de tiempo:___________ |
| \| Si \|  \| No \|  \| \| --- \| --- \| --- \| --- \|  1. Vacunas Queratoconjuntivitis     Marca:__________________ Dosis:____________ Lapso de tiempo:___________ |
| \| Si \|  \| No \|  \| \| --- \| --- \| --- \| --- \|  1. Vacuna Mancha, Gangrena, Enterotoxemia   Marca:__________________ Dosis:____________ Lapso de tiempo:___________ |
| \| Si \|  \| No \|  \| \| --- \| --- \| --- \| --- \|  1. Otras vacunas:   Marca:__________________ Dosis:____________ Lapso de tiempo:___________ |
| \| Si \|  \| No \|  \| \| --- \| --- \| --- \| --- \|  1. Minerales   Principio activo ____________ Marca: __________ Dosis:______ Lapso de tiempo________ |
| 1. Mide temperatura a ingreso de los animales  \| Si \|  \| No \|  \| \| --- \| --- \| --- \| --- \| |
| - ¿Usted realiza una segunda sanidad? ¿Cuántos días después de la primera?   __________________________________________________________________ |
| 1. ¿Realiza **profilaxis** al ingreso de los animales? *(Se define profilaxis con antibióticos a la aplicación de los mismos en forma masiva a todo el rodeo al arribo de los animales)*  \| Si \|  \| No \|  \| \| --- \| --- \| --- \| --- \|   En caso de que su respuesta haya sido afirmativa:   1. Principio activo del ATB utilizado_________ Marca________ Dosis aprox por animal_______ 2. Forma de aplicación (oral-inyectable, etc):   __________________________________________________________________   1. Duración en caso de que sea vía oral:   __________________________________________________________________  __________________________________________________________________   1. ¿Cómo calcula la dosis de aplicación?   __________________________________________________________________   1. ¿Porque aplica profilaxis a los animales?   __________________________________________________________________   1. ¿Cuál es el criterio de selección de los antibióticos?   __________________________________________________________________ |
| \| Si \|  \| No \|  \| \| --- \| --- \| --- \| --- \|  1. ¿Realiza **Metafilaxis**? *(Se define metafilaxis a la aplicación masiva de antibióticos a todo el rodeo que arriba cuando el porcentaje de animales enfermos supera el 15%)*   En caso de que su respuesta haya sido afirmativa:   1. Principio activo del ATB utilizado_________ Marca_________ Dosis aprox por animal______ 2. Forma de aplicación (oral-inyectable, etc):   __________________________________________________________________   1. Duración en caso de que sea vía oral:   __________________________________________________________________   1. ¿Cuál es el criterio de selección de los antibióticos?   __________________________________________________________________   1. ¿Porque realiza metafilaxis a los animales?   __________________________________________________________________   1. ¿Cómo calcula la dosis de aplicación?   __________________________________________________________________ |
| 1. ¿Realiza **tratamientos individuales** al ingreso? Se refiere si se aíslan y tratan en forma individual los animales con signología de enfermedad al arribo.  \| Si \|  \| No \|  \| \| --- \| --- \| --- \| --- \|   En caso de que su respuesta haya sido afirmativa:   1. Principio activo del ATB utilizado________ Marca________ Dosis aprox por animal______ 2. Forma de aplicación (oral-inyectable, etc):   __________________________________________________________________   1. ¿Cuál es el criterio de selección de los antibióticos?   __________________________________________________________________   1. ¿Usted aplica antiinflamatorios esteroides?   Principio activo ____________ Marca__________ Dosis____________  Observaciones (cualquier aclaración que sea de interés, como si se aplica en conjunto con algún antibiótico, con qué objetivo lo aplica, etc)   1. ¿Cuál es el criterio utilizado para tratar a los animales?   __________________________________________________________________   1. ¿Cómo calcula la dosis de aplicación?   __________________________________________________________________ |
| 1. Sanidad en el **proceso de engorde** 2. Mencione si realiza alguna maniobra en particular (más vacunaciones, tratamientos, etc)   __________________________________________________________________   1. Metafilaxis especificar criterio de aplicación. Producto utilizado.   __________________________________________________________________   1. Tratamientos individuales. Especificar 2. Principio activo: Marca: 3. Principio activo: Marca: 4. Principio activo: Marca: 5. Explicar cuál es el criterio de elección para la utilización de cada uno de los antibióticos.   __________________________________________________________________   1. ¿Cuáles son los signos clínicos que considera a la hora de aplicar un tratamiento?   __________________________________________________________________   - ¿Cuánto dura aproximadamente este periodo? ¿Como es la circulación de los animales en el establecimiento?   __________________________________________________________________ |
| 1. Sanidad en el **proceso de terminación** 2. Mencione si realiza alguna maniobra en particular (más vacunaciones, tratamientos, etc)   __________________________________________________________________   1. Metafilaxia: especificar criterio de aplicación. Producto utilizado.   __________________________________________________________________   1. Tratamientos individuales. Especificar 2. Principio activo: Marca: 3. Principio activo: Marca: 4. Principio activo: Marca: 5. Explicar cuál es el criterio de elección para la utilización de cada uno de los antibióticos   __________________________________________________________________   1. ¿Cuáles son los signos clínicos que considera a la hora de aplicar un tratamiento?   __________________________________________________________________ |

| **PATOLOGIAS** |
| --- |

| 1. Principales causas de Morbilidad/Mortalidad   Marzo 2019/ marzo 2020. En base a que menciona dichos datos. ¿Tuvo diagnóstico veterinario? ¿Realiza necropsia a que porcentaje de los animales? ¿Realiza normalmente análisis complementarios para arribar a su diagnóstico presuntivo? O lo que mencionará es estimativo.  **¿Cómo realiza el diagnóstico de las diversas presentaciones?**  __________________________________________________________________ |
| --- |
| Los datos de la siguiente tabla se pueden presentar en números (caso de que el veterinario tenga diagnósticos concretos) o marcando con cruces marcando importancia en su feedlot). Ejemplo una cruz (se presenta esporádicamente), dos cruces, tres cruces (de alta presentación de ese feedlot). |

|  | **MORBILIDAD** | **MORTALIDAD** | **OBSERVACIONES** |
| --- | --- | --- | --- |
| **ENFERMEDADES DE MAYOR CONTAGIO** | | | |
| C Resp Bovino |  |  |  |
| Coccidiosis |  |  |  |
| Sarna |  |  |  |
| Piojos |  |  |  |
| Otros |  |  |  |
| **ERRORES ALIMENTICIOS CON DISFUNCIONES BIOQUÍMICAS** | | | |
| Intoxicación Hídrica |  |  |  |
| Sobrecarga de Rumen – Indigestión por Repleción o Indigestión Simple |  |  |  |
| Otros |  |  |  |
| **POR ERRORES ALIMENTICIOS (cuali-cuantitativos). (Exceso o déficit de un principio alimenticio)** | | | |
| Acidosis láctica aguda |  |  |  |
| Acidosis latente crónica |  |  |  |
| Alcalosis Ruminal. Intoxicación con Urea |  |  |  |
| Enfermedad del Músculo Blanco o Distrofia Muscular Nutricional (Carencia de Selenio y/o Vitamina E) |  |  |  |
| Desbalance Mineral u Osteopatía Nutricional en Bovinos de Recría y Terminación |  |  |  |
| Deficiencia de Zinc en Bovinos. Pododermatitis plantar Proliferativa |  |  |  |
| **POR PROBLEMAS HIGIÉNICOS O CONTAMINACIONES ALIMENTICIAS (Alimentos enmohecidos o alterados - Henos con plantas tóxicas)** | | | |
| Listeriosis Nerviosa |  |  |  |
| Micotoxicosis – Micotoxinas |  |  |  |
| Intoxicación por Sunchillo (Wedelia Glauca- |  |  |  |
| Otras |  |  |  |
| **PATOLOGÍAS POR ERRORES DIETÉTICOS. Distribución irregular (mezclado), Error de dosis** | | | |
| Intoxicación por Monensina |  |  |  |
| Cambio en la formulación |  |  |  |
| Otras |  |  |  |
| **MISCELANEAS** | | | |
| Polioencefalomalacia (PEM) |  |  |  |
| Abscesos Intracraneales |  |  |  |
| Reacciones adversas a la Oxitetraciclina de Larga Acción |  |  |  |
| Golpe De Calor (Estrés calórico – Hipertermia) |  |  |  |
| Fotosensibilización |  |  |  |
| Otras |  |  |  |

| **COMPLEJO RESPIRATORIO BOVINO** |
| --- |

| 1. **Morbilidad (enfermos sobre total) según origen y según categoría** |
| --- |

| Feria |  |
| --- | --- |
| Campo con sanidad conocida (Propio) |  |
| Campo con sanidad conocida (Tercero) |  |
| Campo sanidad desconocida |  |
| Desconocido |  |

| **Categoría** | **Número/Observaciones** |
| --- | --- |
| Ternero/a destete precoz |  |
| Ternero/a destete convencional |  |
| Novillitos 1-2 |  |
| Novillito 2-3 |  |
| Vaquillona 1-2 |  |
| Vaquillona 2-3 |  |
| Vaca |  |
| Toro |  |
| MEJ |  |

| 1. **Mortalidad (muertos sobre total) según origen y según categoría** |
| --- |

| Feria |  |
| --- | --- |
| Campo con sanidad conocida (Propio) |  |
| Campo con sanidad conocida (Tercero) |  |
| Campo sanidad desconocida |  |
| Desconocido |  |

| **Categoría** | **Número/Observaciones** |
| --- | --- |
| Ternero/a destete precoz |  |
| Ternero/a destete convencional |  |
| Novillitos 1-2 |  |
| Novillito 2-3 |  |
| Vaquillona 1-2 |  |
| Vaquillona 2-3 |  |
| Vaca |  |
| Toro |  |
| MEJ |  |

| 1. En qué periodo ocurre la mayor morbilidad/mortalidad.    1. Periodo de adaptación (primeros 30 días)    2. Proceso de engorde    3. Proceso terminación |
| --- |
| 1. ¿Tiene datos de aislamientos bacterianos y antibiogramas? |
| 1. ¿Me podría decir por medio de un ejemplo un caso reciente de Morbilidad/ mortalidad en el establecimiento?  - ¿Qué medidas decidió aplicar? - ¿Por qué decidió realizar ese procedimiento? |

| ALIMENTACIÓN |
| --- |

| 1. Alimentación (detalle con que alimenta a los animales, si pudiera hacerlos por categoría mejor). Proporciones de cada uno de los alimentos y suplementos utilizados 2. Periodo de adaptación (especificación de la duración de este) 3. Proceso de engorde (duración) 4. Proceso terminación (duración) | |
| --- | --- |
| 1. ¿Utiliza materia prima propia para preparar alimento en el establecimiento? ¿Cuál? | |
| 1. Administra ionóforos en el alimento o de alguna otra forma. En caso de que su respuesta sea afirmativa, especifique:      1. Principio activo: …………………. Dosis: …………………………… Marca:……… 2. Forma de administración: …………………………………………………………….. 3. Periodo de engorde: …………………………………………………………………… 4. Periodo de terminación: ……………………………………………………………….. | |
| 1. Administra minerales en el alimento o de alguna otra forma. En caso de que su respuesta sea afirmativa, especifique: 2. Mineral: ………………… Dosis:………….……Forma de administración:…………Periodo de engorde:…………………………………… Periodo de terminación: …………………………………………………………… 3. Mineral: ………………… Dosis:………….……Forma de administración:…………Periodo de engorde:……………………………………Periodo de terminación: …………………………………………………………… 4. Mineral: ………………… Dosis:………….……Forma de administración:…………Periodo de engorde:……………………………………Periodo de terminación: …………………………………………………………… | |
| 1. ¿Administra antibióticos como promotores de crecimiento o en forma preventiva junto con el alimento? En caso de que su respuesta sea afirmativa, especifique   Principio activo: …………………. Dosis: …………………………… Marca:………  Forma de administración: ……………………………………………………………..  Periodo de engorde: ……………………………………………………………………  Periodo de terminación: ……………………………………………………………….. | |
| 1. ¿Administra probióticos junto con el alimento? En caso de que su respuesta sea afirmativa, especifique   Principio activo: …………………. Dosis: …………………………… Marca:………  Forma de administración: ……………………………………………………………..  Periodo de engorde: ……………………………………………………………………  Periodo de terminación: ………………………………………………………………. | |
| 1. ¿Cuánto considera que es la perdida de alimento (viento, formulación, comedero, desperdicio del animal)? |  |

| EFLUENTES |
| --- |

| \| Si \|  \| No \|  \| \| --- \| --- \| --- \| --- \|  1. ¿Posee tratamiento de efluentes?   En caso afirmativo detallar (especificar piletas anaerobias, aerobias, si realizan tratamientos, etc.) |
| --- | --- | --- | --- | --- |
| 1. ¿Cuál es el destino de los efluentes generados? |
| 1. ¿Cada cuánto se realiza la limpieza de los corrales? ¿Cuál es el destino? |
| 1. ¿Cuál es destino de los animales que se mueren en el establecimiento? |
| 1. ¿Cuál es el destino final de los envases y descartes de productos veterinarios? |
| 1. Por favor, describa el sistema de drenaje en los corrales |
| RECURSOS HUMANOS |

| 1. ¿Cuantas personas están afectadas al Feedlot? Especificar los que trabajan de manera directa y/o indirecta |
| --- |
| 1. ¿Tiene veterinario viviendo en el establecimiento? ¿Cuáles son las tareas que debe realizar? |
| 1. ¿Tiene recorredor? ¿Cuáles son sus tareas? |
| 1. ¿Tiene mixero? ¿Cuáles son sus tareas? |
| 1. ¿Tiene personal administrativo afectado a la carga de datos del Feedlot? |
| 1. ¿Tiene nutricionista? ¿Cuál es su tarea y de qué manera está relacionado al Feedlot? Ej. Asesoramiento puntual, visita semanal, mensual. Solo formulación de la dieta o control posterior. |
| 1. ¿Los fines de semana quien alimenta a los animales? ¿Tiene guardia? |
| 1. ¿Cómo realiza la recolección de datos de los tratamientos aplicados? (libro de tratamiento, Excel, etc) |
| \| Si \|  \| No \|  \| \| --- \| --- \| --- \| --- \|  1. ¿Realiza capacitaciones periódicas? |
| 1. Lectura de comederos: |
| 1. Cuando y como las realiza: |
| 1. ¿Sigue alguna escala o parámetro para la lectura? Especifique |
| 1. Cuando realiza el cambio de dieta |
| 1. Limpieza de bebederos: Cuando y como las realiza: |
| 1. Me podría indicar el proceso de selección de antibióticos: ¿en que se basa su decisión para prescribirlos?  - ¿Quién es el encargado de la compra de los productos veterinarios? |
| - ¿Quién hace la indicación para la compra de un producto? |
| ¿Dónde se realizan la compra de los productos veterinarios? |
| - A la hora de decidir comprar un producto usted que es lo que prioriza: (marca, precio) |
| - ¿Me podría dar un ejemplo de cuando usted decidió no aplicar ningún producto? |

**¡¡Muchas gracias por tomarse el tiempo de contestar esta encuesta!!**
