# Supplementary material for: A mixed method approach to analysing patterns and drivers of antibiotic use and resistance in beef farms in Argentina
Source: Front Vet Sci. 2024 Nov 13;11:1454032. doi: 10.3389/fvets.2024.1454032 (PMC11600977; doi:10.3389/fvets.2024.1454032)
Supplement: Supplementary file 1 [file Data_Sheet_1.zip › Document 1.docx]

**"Mapping of antibiotic patterns, use and resistance in the Argentine beef industry"**

*NOTE: The information provided is confidential and anonymous. It will be used EXCLUSIVELY for academic and research purposes and the results obtained from the work will be shared with the collaborators of this study.*

**Contacto:** Dra. Maria Laura Galotta [galottalaura@vet.unicen.edu.ar](mailto:galottalaura@vet.unicen.edu.ar)

| Establishment ID____________________________________________________ |
| --- |
| Role of the interviewee: __________________________________________________ |
| Period **March 2019- March 2020** |

| **GENERAL INFORMATION** |
| --- |

| 1. Number of animals that entered during March 2019- March 2020 |  |
| --- | --- |

| 1. Number of animals sold (or slaughtered) March 2019- March 2020 |  |
| --- | --- |

| 1. Average (Kg) of animals at baseline |  |
| --- | --- |

| 1. Average (Kg) of the finishing animals |  |
| --- | --- |

| 1. Type of Fattening (Select 1 or more options, and indicate in percentage as appropriate) |
| --- |

| Hotel-system |  |
| --- | --- |
| Feedlot fattening |  |
| Early weaned calves |  |
| Pastoral with supplementation |  |

| 1. Origin and number of animals fattened (Select 1 or more options, and indicate percentage as appropriate) |
| --- |

| Market |  |
| --- | --- |
| Farm with known health status (Own) |  |
| Farm with known health status (Third party) |  |
| Farm of unknown health status |  |
| Unknown |  |

| 1. Category that are fattened in the Feedlot (Specify number of animals per cycle): |
| --- |

| **Category** | **Number/Remarks** | **Cycle length** (e.g. Animal Completion Weight/Days/Other) |
| --- | --- | --- |
| Early weaned calves |  |  |
| Conventionally weaned calves |  |  |
| Steers 1-2 years old |  |  |
| Steer 2-3 years old |  |  |
| Heifer 1-2 years old |  |  |
| Heifer 2-3 years old |  |  |
| Cows |  |  |
| Bulls |  |  |
| MEJ (Young Entire Males) |  |  |

| 1. What breeds are present in the establishment? | |
| --- | --- |
|  | - Aberdeen Angus |
|  | - Hereford |
|  | - Braford |
|  | - Brangus |
|  | - Creole |
|  | - Holando Argentino |
|  | - Other |

| 1. With regard to the process of buying animals:  - What region (provinces) of Argentina do they come from?   ______________________________________________________________  ______________________________________________________________   - What would make you change from where you buy animals?   ______________________________________________________________  ______________________________________________________________   - What is the average distance that animals travel to enter the farm?   ______________________________________________________________   - Do you see a greater number of sick animals depending on the origin of the animals? If yes, from which region?   ______________________________________________________________  ______________________________________________________________ |
| --- |

1. What is the final destination of the animals (e.g., domestic consumption or export)?

__________________________________________________________________

| **FACILITIES** |
| --- |

| 1. Total number of pens |  |
| --- | --- |
| 1. Size of the pens   ______________________________________________________________ | |
| 1. Type of soil (material) where the pens are located  \| Earth \|  \| Concrete \|  \| Mixed \|  \| Other \|  \| \| --- \| --- \| --- \| --- \| --- \| --- \| --- \| --- \| | |
| 1. Presence of slopes in the establishment? (If the answer is Yes, indicate where)   ______________________________________________________________ | |
| 1. Could you tell me how the animals are distributed in the pens? (Animal load per pen)   ______________________________________________________________  ______________________________________________________________ | |
| 1. What is the criteria for assembling groups of animals per pen? (e.g.: age, weight, males/females, farm of origin)   ______________________________________________________________ | |
| 1. Are the groups of animals changed during the fattening period? (e.g., slow-growing animals in one group are moved to a later group of younger cattle?)   ______________________________________________________________  ______________________________________________________________ | |
| 1. Is there a hospital pen? If yes, indicate how many animals are currently in that pen.   ______________________________________________________________  ______________________________________________________________ | |
| 1. What criteria is used when deciding to move animals to a hospital pen?   ______________________________________________________________ | |
| 1. Do the animals in this hospital pen, once recovered, return to their original pen, go to another pen or remain in the infirmary until they are sent to slaughter or die?   ______________________________________________________________  ______________________________________________________________ | |
| 1. Are the movement passages and feed passages the same? Do you feel that this causes you any inconvenience in your routine?   ______________________________________________________________ | |
| 1. Where are the water troughs located? (Do pens share troughs)   ______________________________________________________________ | |
| 1. Are there wind-break trees   ______________________________________________________________ | |
| 1. What are the feeders like? (material, design, platform)   ______________________________________________________________ | |
| 1. Is there shade in the pens? (material, location, etc.)   ______________________________________________________________ | |
| \| Yes \|  \| No \|  \| \| --- \| --- \| --- \| --- \|  1. Do you carry out any integrated pest management?   If the answer is Yes, mention the handling you perform:  ______________________________________________________________ | |
| 1. Could you tell me the source of the animals' drinking water (e.g. surface water, groundwater). If it is groundwater, what is the depth of the borehole?   ______________________________________________________________  ________________________________________________________________ | |
| **ANIMAL MANAGEMENT** | |

| 1. What is the form of identification of animals? (eartag, electronic eartag, tattoo, etc)   ______________________________________________________________ | | | | | |
| --- | --- | --- | --- | --- | --- |
| 1. When do you weigh the animals (e.g., on admission, after 4 weeks)?   ______________________________________________________________ | | | | | |
| 1. How many times are animals weighed until they are sold/slaughtered?   ______________________________________________________________ | | | | | |
| 1. Do you use a quarantine pen when the animals enter? | | | | | |
| Yes |  | No |  |  |  |

If Yes, please indicate for how long

___________ days

| 1. **Animal entry health**  - Do you do any stress management? If the answer is Yes, indicate what the handling is.   ______________________________________________________________   - Do you carry out any biosecurity management when the animals enter? If the answer is Yes, indicate when you do it (as soon as the animals enter, after two days)   ______________________________________________________________ |
| --- |

Complete, as appropriate, the health care you perform upon admission:

| \| Yes \|  \| No \|  \| \| --- \| --- \| --- \| --- \|  1. Antiparasitic   Active ingredient ____________ Brand name: __________ Dosage:______ Lapse of tiempo________ |
| --- | --- | --- | --- | --- |
| \| Yes \|  \| No \|  \| \| --- \| --- \| --- \| --- \|  1. Respiratory Disease Vaccines     Brand Name:__________________ Dosage:____________ Time Lapse:___________ |
| \| Yes \|  \| No \|  \| \| --- \| --- \| --- \| --- \|  1. Vaccine for keratoconjuntivitis     Brand Name:__________________ Dosage:____________ Time Lapse:___________ |
| \| Yes \|  \| No \|  \| \| --- \| --- \| --- \| --- \|  1. Vaccines for Blackleg, Gangrene, Enterotoxaemia   Brand Name:__________________ Dosage:____________ Time Lapse:___________ |
| \| Yes \|  \| No \|  \| \| --- \| --- \| --- \| --- \|  1. Other vaccines:   Brand Name:__________________ Dosage:____________ Time Lapse:___________ |
| \| Yes \|  \| No \|  \| \| --- \| --- \| --- \| --- \|  1. Minerals   Active ingredient ____________ Brand name: __________ Dosage:______ Lapse of tiempo________ |
| 1. Do you measure temperature at the entrance of the animals  \| Yes \|  \| No \|  \| \| --- \| --- \| --- \| --- \| |
| - Do you perform a second vaccine dose? How many days after the first one?   ______________________________________________________________ |
| 1. Do you perform **prophylaxis** on entry of the animals? *(Antibiotic prophylaxis is defined as the application of antibiotics to the entire group upon the arrival of the animals)*  \| Yes \|  \| No \|  \| \| --- \| --- \| --- \| --- \|   If your answer was yes:   1. Antibiotic active ingredient used________ Brand________ Approx dose per animal_______ 2. Method of application (oral-injectable, etc.):   ______________________________________________________________   1. Duration in case it is oral:   ______________________________________________________________  _______________________________________________________________   1. How do you calculate the application dose?   _______________________________________________________________   1. Why do you apply prophylaxis to animals?   ______________________________________________________________   1. What is the selection criteria for antibiotics?   ______________________________________________________________ |
| \| Yes \|  \| No \|  \| \| --- \| --- \| --- \| --- \|  1. Do you perform **Metaphylaxis**? *(Metaphylaxis is defined as the massive application of antibiotics to the entire herd that arrives when the percentage of sick animals exceeds 15%)*   If your answer was yes:   1. Active substance of antibiotic used_________ Brand_________ Approx dose per animal______ 2. Method of application (oral-injectable, etc.):   ______________________________________________________________   1. Duration in case it is oral:   ______________________________________________________________   1. What is the selection criteria for antibiotics?   ______________________________________________________________   1. Why does you use metaphylaxis?   ______________________________________________________________   1. How do you calculate the application dose?   _______________________________________________________________ |
| 1. Do you perform **individual treatments** on admission? It refers to whether animals with signs of disease are isolated and treated individually upon arrival.  \| Yes \|  \| No \|  \| \| --- \| --- \| --- \| --- \|   If your answer was yes:   1. Antibiotic active substance use________ Brand________ Approx dose per animal______ 2. Method of application (oral-injectable, etc.):   ______________________________________________________________   1. What is the selection criteria for antibiotics?   ______________________________________________________________   1. Do you apply steroid anti-inflammatory drugs?   Active ingredient ____________ Brand__________ Dose____________  Observations (any clarification that is of interest, such as if it is applied in conjunction with an antibiotic, for what purpose it is applied, etc.)   1. What is the criteria used to treat animals?   ______________________________________________________________   1. How do you calculate the application dose?   ______________________________________________________________ |
| 1. Health in the **fattening process** 2. Mention if you perform any particular procedures (more vaccinations, treatments, etc.)   _______________________________________________________________   1. Metaphylaxis specify application criteria. Product used.   ______________________________________________________________   1. Individual treatments. Specify 2. Active Ingredient: Brand: 3. Active Ingredient: Brand: 4. Active Ingredient: Brand: 5. Explain the criteria of choice for the use of each of the antibiotics.   ______________________________________________________________   1. What are the clinical signs that you consider when applying a treatment?   ______________________________________________________________   - How long does this period last approximately? How is the circulation of animals in the establishment?   ______________________________________________________________ |
| 1. Healing in the **Termination Process** 2. Mention if you perform any particular procedures (more vaccinations, treatments, etc.)   ______________________________________________________________   1. Metaphylaxis: specify application criteria. Product used.   ______________________________________________________________   1. Individual treatments. Specify 2. Active Ingredient: Brand: 3. Active Ingredient: Brand: 4. Active Ingredient: Brand: 5. Explain the criteria of choice for the use of each of the antibiotics   ______________________________________________________________   1. What are the clinical signs that you consider when applying a treatment?   ______________________________________________________________ |

| **PATHOLOGIES** |
| --- |

| 1. Main causes of Morbidity/Mortality   March 2019/ March 2020. Did you have a veterinary diagnosis? Do you perform necropsy on what percentage of the animals? Do you normally perform complementary tests to arrive at your presumptive diagnosis? Or is it a guess.  **How do you diagnose the various presentations?**  __________________________________________________________________ |
| --- |
| The data in the following table can be presented in numbers (if the veterinarian has specific diagnoses) or marked with crosses marking importance on their feedlot). Example one cross (occurs sporadically), two crosses, three crosses (high presentation of that feedlot). |

|  | **MORBIDITY** | **MORTALITY** | **REMARKS** |
| --- | --- | --- | --- |
| **MAJOR CONTAGIOUS DISEASES** | | | |
| BRD |  |  |  |
| Coccidiosis |  |  |  |
| Scabies |  |  |  |
| Head lice |  |  |  |
| Other |  |  |  |
| **FEEDING ISSUES WITH BIOCHEMICAL DYSFUNCTIONS** | | | |
| Water Intoxication |  |  |  |
| Rumen Overload – Indigestion by Repletion or Simple Indigestion |  |  |  |
| Other |  |  |  |
| **DUE TO FEEDING ERRORS (qualitative-quantitative). (Excess or deficit of a principle food)** | | | |
| Acute lactic acidosis |  |  |  |
| Chronic latent acidosis |  |  |  |
| Ruminal alkalosis. Urea Poisoning |  |  |  |
| White Muscle Disease or Nutritional Muscular Dystrophy (Selenium and/or Vitamin E Deficiency) |  |  |  |
| Mineral Imbalance or Nutritional Osteopathy in Rearing and Finishing Cattle |  |  |  |
| Zinc deficiency in cattle. Proliferative Plantar Pododermatitis |  |  |  |
| **DUE TO HYGIENIC PROBLEMS OR FOOD CONTAMINATION (Moldy or altered food - Hay with toxic plants)** | | | |
| Listeriosis Nerviosa |  |  |  |
| Mycotoxicosis – Mycotoxins |  |  |  |
| Sunchillo poisoning (Wedelia Glauca- |  |  |  |
| Other |  |  |  |
| **PATHOLOGIES DUE TO DIETARY ERRORS. Irregular distribution (mixed), Dose error** | | | |
| Monensin poisoning |  |  |  |
| Change in formulation |  |  |  |
| Other |  |  |  |
| **MISCELLANEOUS** | | | |
| Polioencefalomalacia (PEM) |  |  |  |
| Intracranial Abscesses |  |  |  |
| Adverse Reactions to Long-Acting Oxytetracycline |  |  |  |
| Heat stroke (heat stress – hyperthermia) |  |  |  |
| Photosensitization |  |  |  |
| Other |  |  |  |

| **BOVINE RESPIRATORY COMPLEX** |
| --- |

| 1. **Morbidity (patients over total) by origin and category** |
| --- |

| Market |  |
| --- | --- |
| Farm with known health status (Own) |  |
| Farm with known health status (Third party) |  |
| Farm of unknown health status |  |
| Unknown |  |

| **Category** | **Number/Remarks** |
| --- | --- |
| Early weaned calves |  |
| Conventionally weaned calves |  |
| Steers 1-2 years old |  |
| Steer 2-3 years old |  |
| Heifer 1-2 years old |  |
| Heifer 2-3 years old |  |
| Cows |  |
| Bulls |  |
| MEJ (Young Entire Males) |  |

| 1. **Mortality (deaths over total) by origin and by category** |
| --- |

| Market |  |
| --- | --- |
| Farm with known health status (Own) |  |
| Farm with known health status (Third party) |  |
| Farm of unknown health status |  |
| Unknown |  |

| **Category** | **Number/Remarks** |
| --- | --- |
| Early weaned calves |  |
| Conventionally weaned calves |  |
| Steers 1-2 years old |  |
| Steer 2-3 years old |  |
| Heifer 1-2 years old |  |
| Heifer 2-3 years old |  |
| Cows |  |
| Bulls |  |
| MEJ (Young Entire Males) |  |

| 1. In which period the greatest morbidity/mortality occurs.    1. Adaptation period (first 30 days)    2. Fattening process    3. Termination Process |
| --- |
| 1. Do you have data on bacterial isolates and antimicrobial susceptibility testing? |
| 1. Could you tell me by means of an example a recent case of Morbidity/Mortality in the facility?  - What measures did you decide to apply? - Why did you decide to perform this procedure? |

| FEEDING |
| --- |

| 1. Feeding (details of feeding regimen, by category). Proportions of each of the foods and supplements used 2. Adaptation period (specifying the duration of the period) 3. Fattening process (duration) 4. Process Termination (Duration) | |
| --- | --- |
| 1. Do you use your own raw materials to prepare food on the premises? Which one? | |
| 1. Administer ionophores into food or in some other way. If yes, please specify:      1. Active ingredient: ...................... Dose:................................. Brand:......... 2. Method of administration: ....................................................................... 3. Fattening period: .............................................................................. 4. Termination Period: .......................................................................... | |
| 1. Administer minerals in food or in some other way. If yes, please specify: 2. Mineral:..................... Dose:................... Method of administration:............ Fattening period:.......................................... Termination period: ..................................................................... 3. Mineral:..................... Dose:................... Method of administration:............ Fattening period:.......................................... Termination period: ..................................................................... 4. Mineral:..................... Dose:................... Method of administration:............ Fattening period:.......................................... Termination period: ..................................................................... | |
| 1. Do you administer antibiotics as growth promoters or preventively along with food? If yes, please specify   Active ingredient: ...................... Dose:................................. Brand:.........  Method of administration: .......................................................................  Fattening period: ..............................................................................  Termination Period: .......................................................................... | |
| 1. Do you give probiotics along with food? If yes, please specify   Active ingredient: ...................... Dose:................................. Brand:.........  Method of administration: .......................................................................  Fattening period: ..............................................................................  Termination Period: ......................................................................... | |
| 1. How much do you consider to be the loss of feed (wind, formulation, feeder, animal waste)? |  |

| EFFLUENT |
| --- |

| \| Yes \|  \| No \|  \| \| --- \| --- \| --- \| --- \|  1. Do you have a manure treatment plant?   If so, detail (specify anaerobic pools, aerobic pools, if they perform treatments, etc.) |
| --- | --- | --- | --- | --- |
| 1. What happens to the manure? |
| 1. How often are the corrals cleaned? Where does the waste go? |
| 1. What is the fate of the animals that die in the establishment? |
| 1. What is the final destination of the packaging and discards of veterinary products? |
| 1. Please describe the drainage system in the pens |
| HUMAN RESOURCES |

| 1. How many people are affected by the Feedlot? Specify those that work directly and/or indirectly |
| --- |
| 1. Does he have a veterinarian living in the facility? What are the tasks you need to perform? |
| 1. Do you have a runner? What are their tasks? |
| 1. Do you have a mixer? What are their tasks? |
| 1. Do you have administrative staff affected by the Feedlot's data upload? |
| 1. Do you have a nutritionist? What is your task and how is it related to the Feedlot? E.g. punctual advice, weekly or monthly visits. Only formulation of the diet or subsequent control. |
| 1. On weekends, who feeds the animals? Do you have a guard? |
| 1. How do you collect data on the treatments applied? (treatment book, Excel, etc) |
| \| Yes \|  \| No \|  \| \| --- \| --- \| --- \| --- \|  1. Do you conduct regular training? |
| 1. Mixer wagon scales: |
| 1. When and how they are performed: |
| 1. Do you follow any callibration? Specify |
| 1. When you make the change in diet |
| 1. Cleaning of drinking fountains: When and how to do them: |
| 1. Could you tell me about the process of selecting antibiotics: what is your decision to prescribe them based on?  - Who is in charge of purchasing veterinary products? |
| - Who makes the indication for the purchase of a product? |
| Where are veterinary products purchased? |
| - When deciding to buy a product, what do you prioritize: (brand, price) |
| - Could you give me an example of when you decided not to apply any products? |

**Thank you so much for taking the time to take this survey!!**
